# Large-Scale Facile Synthesis of Biomass Fibers and High-Entropy Metal Hierarchical Porous Carbon toward Enhanced Electromagnetic Absorption

**DOI:** 10.34133/research.0868

**Published:** 2025-09-16

**Authors:** Peiyu Cui, Pengbo Zou, Yifan Kang, Xiang Yan, Xin Zhou, BoKun Wang, Fan Wu, Shibing Pan, Jiacheng Ma, Wenhuan Huang

**Affiliations:** ^1^ Key Laboratory of Chemical Additives for China National Light Industry, College of Chemistry and Chemical Engineering, Shaanxi University of Science and Technology, Xi’an 710021, China.; ^2^ Department of Chemistry, School of Science, Tianjin University, Tianjin 300072, China.; ^3^ Shandong Nonmetallic Material Institute, Jinan 250031, China.

## Abstract

The elevated dielectric properties of carbonized cotton fibers with refined conductive networks result in substantial impedance mismatch, severely compromising their electromagnetic wave (EMW) absorption performance. Using cotton fibers and [Zn(pz)_2_]*_n_* complexes as easily prepared and low-cost raw materials, nano-hierarchically porous MnFeCuCe@C composites were constructed efficiently through optimization of the dynamic balance of element content and carbonization temperature. The results show that MnFeCuCe@C-20% exhibited a minimum reflection loss (RL_min_) of −81.44 dB with a thickness of only 1.52 mm at an ultralow loading of 20 wt%, and the effective absorption bandwidth of MnFeCuCe@C-900 °C was also remarkably enlarged to 5.13 GHz at a thickness of 1.6 mm. Modifying only the metal content of the precursor and carbonization temperature can effectively reinforce the magnetic–dielectric synergy and impedance matching. The augmented EMW attenuation primarily stems from the inherent hierarchical porous structure of the MnFeCuCe@C nanocomposite and the rapid electron migration within its high-entropy metal particles. Furthermore, its excellent dissipation capability in practical application scenarios was demonstrated further through radar cross-section simulations. This work comprehensively delineates the optimization strategies for material dielectric properties as well as the convenient and environmental synthesis method.

## Introduction

Electromagnetic waves (EMWs), serving as the cornerstone for both energy transmission and information transfer, are extensive applied in diverse fields including radar detection, medical imaging, and aerospace [1–4]. However, electromagnetic interference (EMI) pollution has become increasingly severe due to the proliferation of overlapping electronic devices, posing a threat to human health and information security [5–8]. Furthermore, electronic security is now equally critical as national defense security. In modern information warfare, military weaponry or electronic reconnaissance equipment rendered inoperative due to EMI or shielding would likely result in a catastrophic loss of combat capability [9,10]. Therefore, to address the critical challenges of EMI and radiation, the development of highly efficient EMW absorbers has emerged as an imperative research mission for scientists and engineers [11–13].

Biomass fibrous materials, as sustainable raw materials, were utilized widely for the fabrication of lightweight functional materials, attributable to 2 primary factors: Firstly, the inherent advantages of the raw material itself, including low cost, low density, natural renewability, and an abundance of active sites (N and O elements) [14–18]. Secondly, carbon materials derived from biomass retain natural morphological characteristics, and properties such as porous structure, low density, high specific surface area, and abundant delocalized π electrons endow the composite materials with excellent flexibility and scalability [19–22]. However, when carbon fibers directly were employed as EMW absorbers, it exhibits severe impedance mismatch due to their surface-abundant electron transport pathways, which could be related to the high dielectric properties. Furthermore, only a small portion of incident EMWs is rapidly dissipated, resulting in a high attenuation capability. The lack of magnetic loss, coupled with the imbalance between impedance matching and attenuation capability, severely limits the EMW absorption performance of absorbers.

Strategically designing heterostructures incorporating high-entropy metal particles and carbon fibers can enhance the defect density within carbon fibers, which enhances the boosted polarization relaxation effects within composite materials [23–30]. Recently, metal-organic frameworks (MOFs) can serve as an effective medium for compounding with carbon fibers. Techniques such as hydrothermal methods [31–33], pyrolysis [34–37], and laser-assisted carbonization [38] enable the uniform deposition of high-entropy metal particles onto the carbon fibers surface. For example, Jin et al. [31] prepared NiCo@NPC@CF-x composite samples derived from Ni/Co-MOF particles and biomass cotton by simple hydrothermal methods and the carbonization process. The hierarchical NiCo@NPC@CF achieves a minimum reflection loss (RL_min_) of −46.5 dB and an effective absorption bandwidth (EAB) of 10.0 GHz at a thickness of 3.5 mm, suggesting that the substantial enhancement in wave absorption performance stems primarily from synergistic contributions of the hierarchical pore structure. However, it remains highly challenging to controllably design metallic carbon materials with both effective magnetic and dielectric losses on natural fiber-based substrates while simultaneously achieving tunable impedance matching.

In this work, we innovatively utilize the abundant active sites on cotton fiber surfaces to in situ grow [Zn(pz)_2_]*_n_* complexes (MOF). The introduction of the MOF efficiently enhances the interfacial interactions between metal salts and cotton fiber components. By precisely controlling element content and carbonization temperature, the integration of high-entropy metal particles within the carbon fiber matrix enhances the magnetic–dielectric synergy of the material. This approach successfully constructs MnFeCuCe@C composites exhibiting high EMW response characteristics. Notably, the MnFeCuCe@C-20% composite achieves a remarkable reflection loss of −81.44 dB at a minimal filler loading of only 20 wt% in the paraffin matrix, with a maximum absorption bandwidth of 5.12 GHz (achieved at 900 °C) and a thin matching thickness of 1.6 mm. Furthermore, the radar cross-section (RCS) reduction reaches 31.72 dB·m^2^. These results provide a reasonable explanation for the EMW absorption mechanism in MnFeCuCe@C composites. The material simultaneously maintains optimized attenuation capability and excellent impedance matching, establishing an effective platform for balancing conduction loss and relaxation loss mechanisms. The low cost of cotton and the [Zn(pz)_2_]*_n_* complexes, combined with the simple synthesis process, offers a novel and highly efficient strategy for designing high-performance EMW absorbers.

## Results and Discussion

### Assembly of high-entropy porous MnFeCuCe@C EMW absorbing materials

**Fig. 1.**
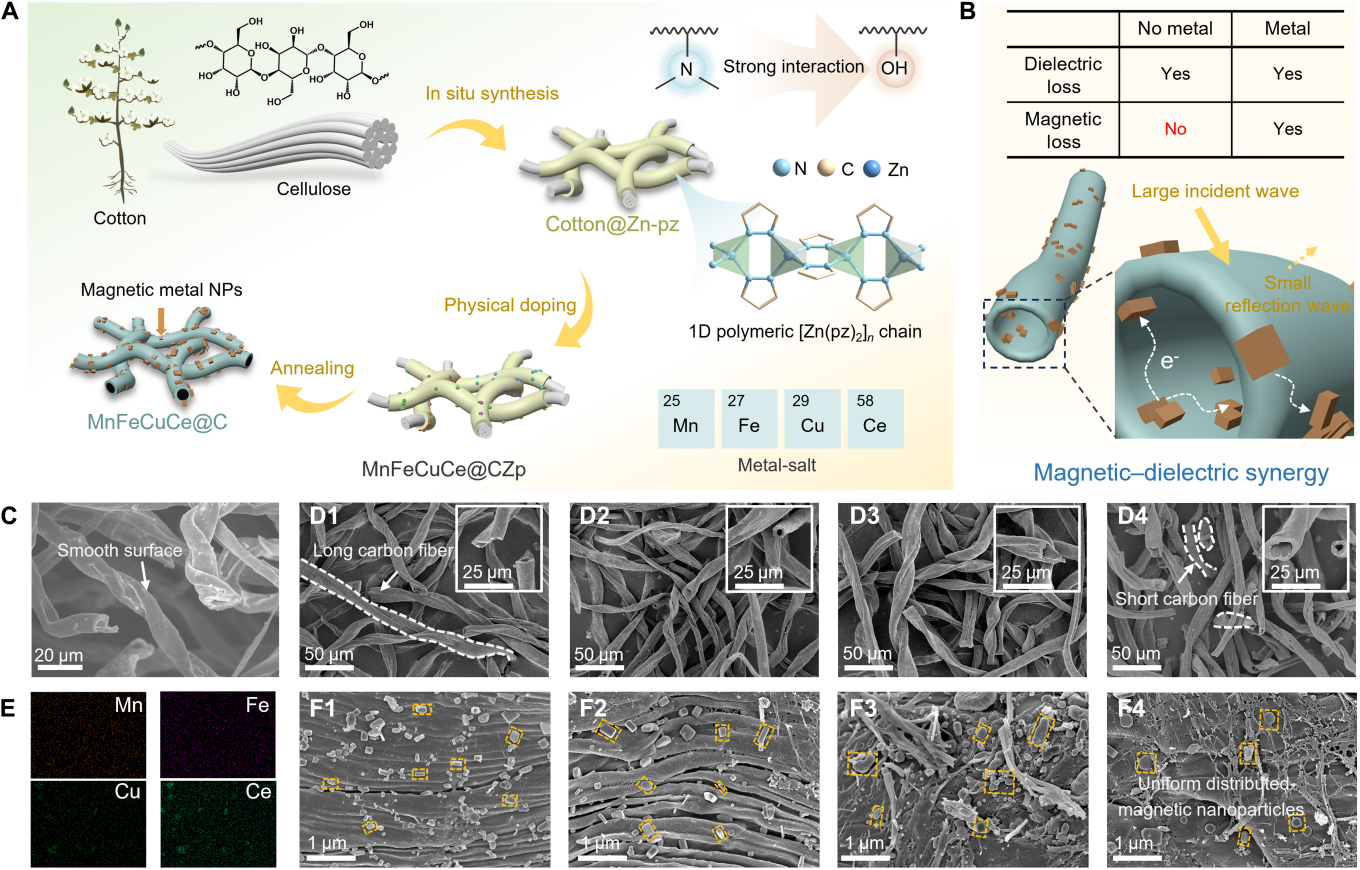
(A) Schematic of the synthetic procedure for MnFeCuCe@C composites for efficient electromagnetic wave absorption. (B) Magnetic–dielectric synergy of MnFeCuCe@C composites. (C) SEM images for cotton fiber after carbonation. SEM images for (D1 and F1) MnFeCuCe@C-700 °C, (D2 and F2) MnFeCuCe@C-800 °C, (D3 and F3) MnFeCuCe@C-900 °C, and (D4 and F4) MnFeCuCe@C-1,000 °C. (E) The elemental mapping of MnFeCuCe@C-1,000 °C.

Natural cotton fibers, as EMW absorbing carbon materials, exhibit a microscale hollow tube-like structure. However, due to the abundant dielectric properties inherent in carbon fiber surfaces, the majority of incident EMWs are reflected at the interface and cannot effectively penetrate the carbon fiber interior, resulting in material impedance mismatch. In contrast, high-entropy metal particles can tune the dielectric properties of carbon fibers and introduce magnetic dissipation, thereby enhancing the EMW dissipation performance of nanocomposites. Simultaneously, as one-dimensional polymeric chain MOFs, [Zn(pz)_2_]*_n_* complexes feature accessible active sites. This characteristic renders them exceptionally suitable for achieving uniform loading of high-entropy metal particles onto the carbon material’s surface through in situ growth and pyrolysis processes (Fig. 1B).

The Cotton@Zn-pz composites were prepared by growing [Zn(pz)_2_]*_n_* complexes in situ on the cotton fiber surfaces at room temperature. However, no peaks corresponding to [Zn(pz)_2_]*_n_* crystal appeared in the x-ray powder diffraction (XRD) pattern, primarily due to trace deposits on cotton surfaces (Fig. S2A). Moreover, the standard card confirmed that the synthesized white powder was the [Zn(pz)_2_]*_n_* complex. Subsequently, Cotton@Zn-pz was immersed and fully infiltrated in a methanol solution containing multielement metal salts. Tailored MnFeCuCe@C nanocomposites were then obtained through thermal treatment of MnFeCuCe@CZp at 700, 800, 900, or 1,000 °C under N_2_ atmosphere. A highly convenient and green synthetic method is clearly demonstrated in Fig. 1A. Scanning electron microscope (SEM) images of carbon fibers (Fig. 1C) show that they remarkably retained their original smooth surfaces and hollow tube-like structures after calcination. Figure 1D1 to D4 depict SEM images of MnFeCuCe@C-20% composites treated at different carbonization temperatures. MnFeCuCe@C-700 °C, MnFeCuCe@C-800 °C, MnFeCuCe@C-900 °C, and MnFeCuCe@C-1,000 °C maintained the hollow fibrous morphology. However, pronounced surface wrinkling occurred on the carbonized cotton fibers, attributable to synergistic effects from substantial organic ligands mass loss and bending deformation during thermal processing. The MnFeCuCe@C-1,000 °C nanocomposites exhibited extensive fracturing, exposing more mesoporous structures. This architectural evolution facilitates efficient EMW penetration into the material matrix, enhancing EMW dissipation mechanisms. Due to evaporation and decomposition of organic ligands within the [Zn(pz)_2_]*_n_* complexes, minimal carbon networks were retained while abundant cubic metal nanoparticles became anchored onto the carbon fiber surfaces, as shown in Fig. 1F1 to F4. Elemental mapping of MnFeCuCe@C-1,000 °C nanocomposites (Fig. 1E) confirms the homogeneous dispersion of high-entropy metal particles across the carbon fiber surfaces (Fig. S1). Collectively, these results confirm the successful fabrication of MnFeCuCe@C composites through a facile and environmentally friendly synthetic route.

The crystal structure and the phase component for as-synthesized MnFeCuCe@C samples were studied by XRD analysis. As shown in Fig. S2B, pristine carbon fibers exhibit 2 broad, weak diffraction peaks at ~22° and ~42°, ascribed to the (002) and (101) planes of graphitic carbon, respectively [39]. For MnFeCuCe@C materials, the feeble (002) diffraction peak at 22° was observed, indicating that these composites maintain the basic structure of carbon fiber matrix. Concurrently, XRD analysis reveals diminished intensity of the graphite-like characteristic peaks in MnFeCuCe@C materials (Fig. S2C). This reduction occurs for 2 reasons: On the hand, increased metal content impedes carbon ordering, resulting in higher defect density; on the other hand, elevated annealing temperature partially disrupts the graphitic structure. This disruption is a critical factor for enhancing the magnetic loss capabilities inherent to pristine carbon fibers. The primary diffraction peaks corresponding to the magnetic phases in MnFeCuCe@C materials strengthens markedly, with no evidence of phase transformation. This confirms the successful in situ anchoring of magnetic metal nanoparticles onto the carbon fibers.

**Fig. 2.**
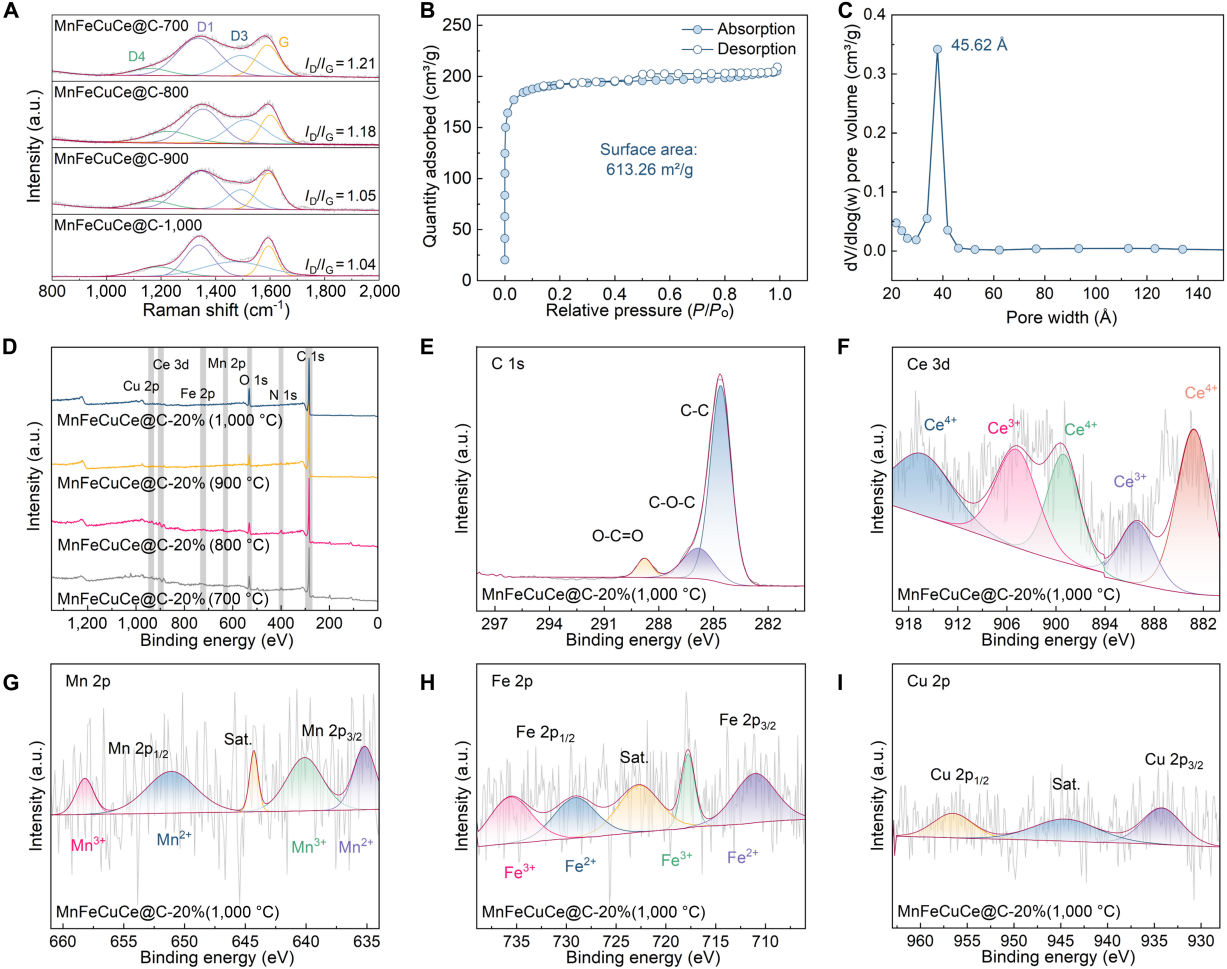
(A) Raman spectra of MnFeCuCe@C-700 °C, MnFeCuCe@C-800 °C, MnFeCuCe@C-900 °C, and MnFeCuCe@C-1,000 °C. (B and C) Nitrogen adsorption–desorption isotherms of MnFeCuCe@C-1,000 °C. (D) The XPS spectra of MnFeCuCe@C-700 °C, MnFeCuCe@C-800 °C, MnFeCuCe@C-900 °C, and MnFeCuCe@C-1,000 °C. (E) C 1s spectrum, (F) Ce 3d spectrum, (G) Mn 2p spectrum, (H) Fe 2p spectrum, and (I) Cu 2p spectrum of MnFeCuCe@C-1,000 °C.

The degree of graphitization in cotton-derived fiber critically determines the conductive loss capabilities of MnFeCuCe@C materials. The ratio (*I*_D_/*I*_G_), which serves as an indicator of structural disorder, illustrates that the MnFeCuCe@C-1,000 °C absorber possesses a high degree of graphitization. Increasing carbonization temperature promotes structural disordering and elevates defect density within the MnFeCuCe@C materials. The role of cotton fibers in MnFeCuCe@C composites extends beyond providing carbon fibers; it also facilitates the formation of abundant oxygen-containing functional groups, as shown in Fig. 2E and Fig. S3E1 to E3; distinct peaks located at 284.6, 285.8, and 288.8 eV correspond to C-C, C-O-C, and O-C=O, respectively. Polar centers generated by these carbon-oxygen functional groups enhance dipole relaxation within the MnFeCuCe@C materials, thereby promoting electromagnetic energy dissipation. Furthermore, N_2_ adsorption/desorption isotherms of MnFeCuCe@C-1,000 °C at 77 K (Fig. 2B) exhibit typical type IV isotherms with obvious hysteresis loops, indicating mesoporous structure. The calculated Brunauer–Emmett–Teller-specific surface area is 613.26 m^2^/g, and Fig. 2C indicates a primary mesopore diameter of 45.62 Å. This uniformly distributed mesoporous structure of MnFeCuCe@C-1,000 °C ensures substantial dissipation of incident EMWs within its interior.

To further investigate the phase composition and valence states of the MnFeCuCe@C nanocomposites, x-ray photoelectron spectroscopy (XPS) was performed (Fig. 2D). The full survey spectrum reveals 7 distinct peaks at binding energies of 283.7, 399.5, 531.3, 626.4, 723.6, 901.5, and 936.0 eV, assigned to C 1s, N 1s, O 1s, Mn 2p, Fe 2p, Ce 3d, and Cu 2p signals, respectively. Figure 2F depicts the Ce 3d spectrum, which deconvolutes into characteristic peaks for mixed valence states: peaks at 886.2 and 905.1 eV correspond to Ce^3+^, while peaks at 882.2, 900.1, and 915.1 eV correspond to Ce^4+^ [40,41]. The result of Mn 2p resolution is given in Fig. 2G, which indicates that the peaks at 635.1 and 651.1 eV correspond to Mn^2+^, and the peaks at 640.1 and 658.1 eV are classified as Mn^3+^ (Fig. S3A1 to A3). Similarly, the Fe 2p XPS spectra (Fig. 2H and Fig. S3B1 to B3) exhibit peaks at 710.9 and 728.9 eV characteristic of Fe^2+^, while peaks at 717.7 and 735.4 eV correspond to Fe^3+^. As shown in Fig. 2I and Fig. S3C1 to C3, the high-resolution Cu 2p spectrum shows 2 main peaks at 934.2 and 956.6 eV, which are assigned to Cu 2p3/2 and Cu 2p1/2, respectively. Briefly, this coexistence of multiple oxidation states originates from rapid nucleation kinetics at ultrahigh temperatures, facilitating simultaneous stabilization of diverse metal valences within anisotropic nanostructures. Multivalent metals enhance electron transfer dynamics within high-entropy metal particles and promote extensive multiphase interface formation. This magnetic–dielectric synergy efficiently augments EMW absorption and scattering in the MnFeCuCe@C nanocomposites.

### The influencing factors of the customized EM response patterns: Carbonization temperatures and metal content

**Fig. 3.**
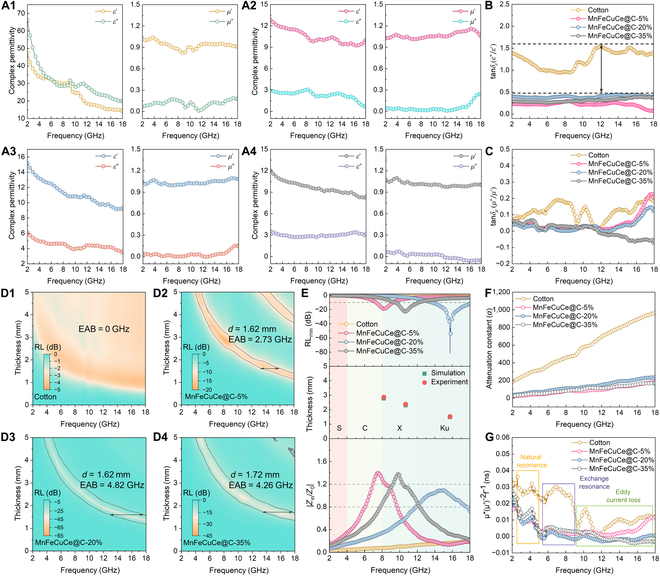
(A1 to A4) Complex permittivity and permeability of cotton, MnFeCuCe@C-5%, MnFeCuCe@C-20%, and MnFeCuCe@C-35%, respectively. (B) Dielectric loss tangents (tan *δε*) and (C) magnetic loss tangents (tan *δμ*). (D1 to D4) 2D RL contour maps for cotton, MnFeCuCe@C-5%, MnFeCuCe@C-20%, and MnFeCuCe@C-35%, respectively. (E) RL/(λ/4)/*Z*–*f* curve. (F) Attenuation constant (*α*). (G) *C*_0_–*f* curve.

Because of the uniform incorporation of magnetic components to the dielectric carbon fiber, magnetic loss plays a key role in the improving the impedance mismatch dominated by the high dielectric behavior in the carbon fiber. Complex permittivity and permeability are measured to further study the EMW absorption properties of MnFeCuCe@C [42]. In Fig. 3A1 to A4, the real part (*ε′*) and imaginary part (*ε″*) of cotton-derived carbon fiber permittivity range from 66 to 17 and from 47 to 14, respectively, while the real part (*ε′*) of MnFeCuCe@C sample permittivity ranges from 13 to 10, from 15 to 9, and from 12 to 8, respectively, and the imaginary part (*ε″*) of MnFeCuCe@C sample permittivity ranges from 3 to 0.7, from 6 to 3.5, and from 3.5 to 3, respectively. In accordance with the experimental findings, the real part of the permittivity (*ε’*) of all samples exhibits a decline with an increase in frequency. This phenomenon manifests as a distinctive scattering signature, predominantly originating from the enhancement of polarization lag induced by high-frequency electric field oscillations [43]. Furthermore, as shown in Fig. 3B, the dielectric dissipation can be quantified by the tangent of dielectric losses (tan *δε* = *ε″*/*ε’*). The tan *δε* values of all samples decreased from 1.5 to 0.4 with the increase of metal content [44]. Based on the Debye formulation (Eqs. S3 to S6), the Cole–Cole curves offer a more intuitive means of evaluating the dielectric behavior of MnFeCuCe@C samples, where the semicircle and straight line represent the polarization relaxation (*ε*_p_″) and the conduction loss (*ε*_c_″), respectively. As shown in Fig. S4, MnFeCuCe@C-20% nanocomposites exhibit a more pronounced semicircle, demonstrating its superior dipole polarization loss. This can be attributed to the high-entropy metal in MnFeCuCe@C disrupting the equilibrium of charge distribution, leading to an inhomogeneous charge distribution. This distribution can induce the oriented alignment of dipoles, thereby generating dielectric properties similar to those of interfacial polarization. This characteristic helps to enhance the absorption efficiency of MnFeCuCe@C for EMWs. Moreover, the intensified dipole polarization also promotes the effective dissipation of EMW energy. Figures S5 and S6 indicate that 3 MnFeCuCe@C samples with similar conductivity may exhibit similar conduction loss. However, the *ε*_p_″ curve of carbon fibers in the 2 to 18 GHz frequency range is notably higher than 3 other samples; this disparity arises because the MnFeCuCe nanoparticles partition the continuous conductive network established by the carbon fiber. Furthermore, metal elemental loading and the structure of [Zn(pz)_2_]*_n_* complexes influence electron mobility, resulting in the reconfigured conductive loss that synergistically complements the magnetic loss [45,46]. As can be seen, the real part (*μ′*) and imaginary part (*μ″*) of the permeability for all samples remain relatively consistent within the tested frequency range. Furthermore, in all MnFeCuCe@C composites, the tan *δε* values are greater than tan *δμ* (*μ″*/*μ′*), confirming dielectric loss dominance in electromagnetic absorption (Fig. 3C).

In Fig. 3D1 to D4, as well as Eqs. S1 and S2 and Figs. S7 and S8, the electromagnetic parameters of MnFeCuCe@C composites were measured at a filler content of 20 wt%. It can be found that metal salt content determines the EMW dissipation capacity of MnFeCuCe@C composites within a certain range. Among them, the minimal reflection loss (RL_min_) of MnFeCuCe@C-5% is only −18.95 dB at a thickness of 2.86 mm, and MnFeCuCe@C-20% is the optimal absorber (with an RL_min_ value of −81.44 dB at a thickness of 1.52 mm and an EAB ranging from 13.7 to 18.0 GHz at a thickness of 1.62 mm). A further increase in the metal salt content of the precursor to 35% led to a deterioration in EMW absorption performance, the RL_min_ value was −23.10 dB at a thickness of 2.37 mm, and the EAB was 4.26 GHz at a thickness of 1.72 mm. Furthermore, the tan𝛿𝜇 values of both MnFeCuCe@C-5% and MnFeCuCe@C-20% exhibited a substantial increase in the high-frequency region. In contrast, MnFeCuCe@C-35% demonstrated an abnormally low tan𝛿𝜇 value, which is attributed to the agglomeration of metal nanoparticles on the carbon fiber surfaces, creating additional reflection sites; these sites prevented effective EMW absorption. Nevertheless, these findings also indicate that incorporating magnetic metals remains an effective strategy for enhancing the EMW dissipation capability of the material. Additionally, both dielectric loss and magnetic loss collectively govern the overall EMW attenuation capacity of MnFeCuCe@C composites.

According to the quarter-wavelength (λ/4) matching theory (Fig. 3E), the slight discrepancy observed between the experimental and simulated matching thickness (*t*_m_) values can be attributed to the marked frequency dependence of polarization relaxation processes [2]. Impedance matching, defined as the degree of matching between the input impedance of absorbers and the impedance of free space, is a prerequisite for achieving strong EMW absorption. The impedance matching ratio of the MnFeCuCe@C-20% absorber, within the optimal range of 0.8 to 1.2, provides enhanced EMW dissipation capability, as evidenced in Figs. S9 and S10. The attenuation constant (*α*) value represents another critical factor for evaluating the performance of high-efficiency absorbers (Eq. S7). As shown in Fig. 3F, pristine carbon fibers exhibit a high attenuation constant. However, this occurs alongside severe impedance mismatch caused by their highly dielectric nature, resulting in important reflection of incident EMWs; only a small fraction is instantaneously absorbed and dissipated upon surface contact. In contrast, the MnFeCuCe@C-20% absorber achieves a synergistic balance between impedance matching and attenuation capability, enabling sustained dissipation of a greater proportion of incident EMW. The *C*_0_ curves of the as-prepared samples, displayed in Fig. 3G (Eq. S8), further elucidate the magnetic loss mechanisms in MnFeCuCe@C composites. The MnFeCuCe@C samples exhibit marked fluctuations within the 2 to 5 GHz range, primarily due to exchange resonance. In the higher frequency region (10 to 18 GHz), the *C*_0_ values demonstrate minimal variation, indicating the dominance of eddy current losses in contributing to the magnetic dissipation of MnFeCuCe@C composites. Furthermore, minor resonance peaks observed in the mid-frequency range (5 to 9 GHz) are primarily associated with exchange resonance effects among the magnetic particles.

**Fig. 4.**
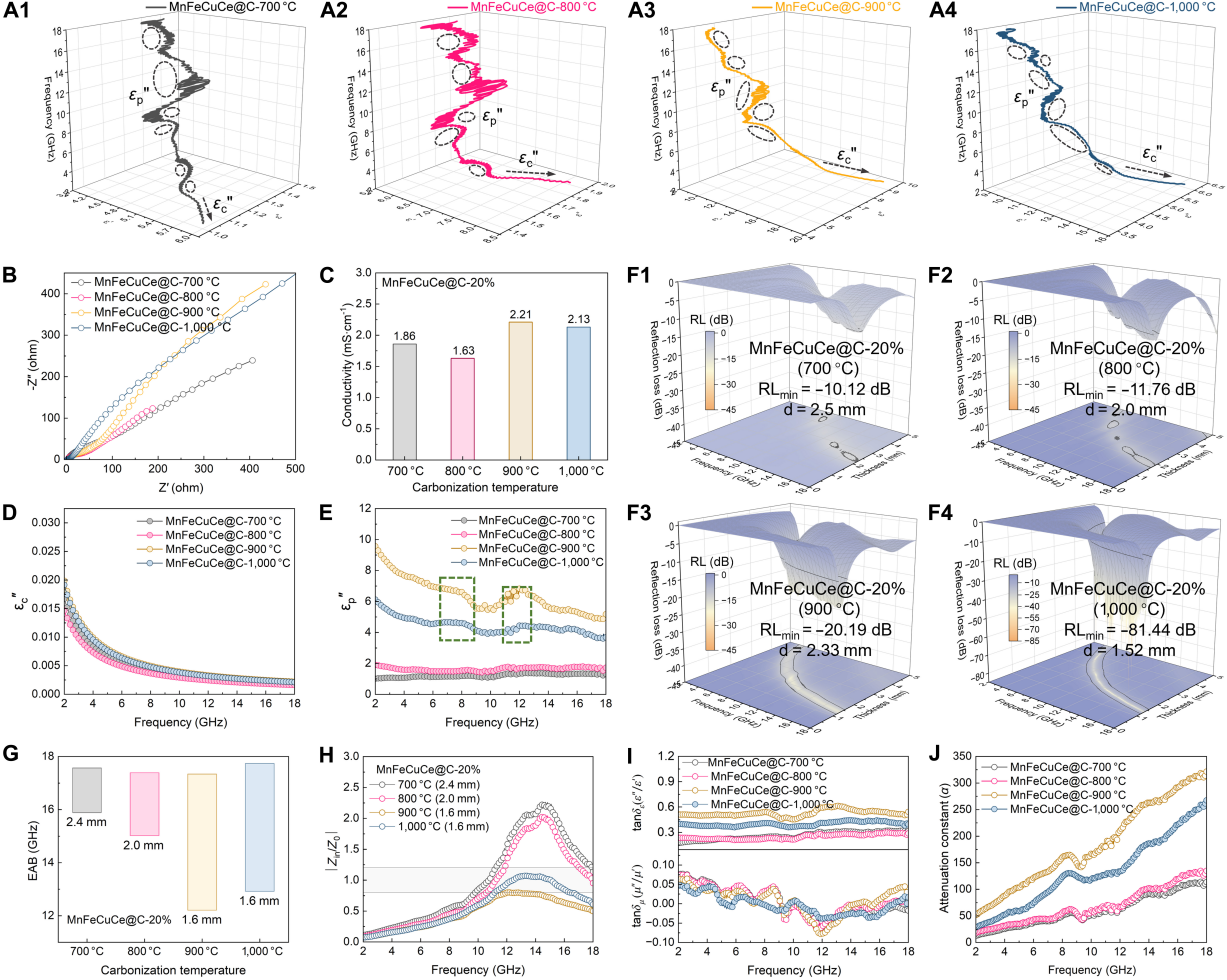
3D Cole–Cole curves (*ε′*–*ε″–f* plots) for (A1) MnFeCuCe@C-700 °C, (A2) MnFeCuCe@C-800 °C, (A3) MnFeCuCe@C-900 °C, and (A4) MnFeCuCe@C-1,000 °C. (B) Nyquist plots. (C) Electrical conductivity. (D) Conduction loss. (E) Polarization loss. (F1 to F4) EWA properties. (G) Summary of EAB and RL_min_ for the samples. (H) The |*Z*_in_/*Z*_0_| plots. (I) Dielectric loss tangents (tan *δε*) and magnetic loss tangents (tan *δμ*). (J) Attenuation constant (*α*).

In order to deeply analyze the critical triggering factors governing the customized electromagnetic response patterns, MnFeCuCe@C-700 °C, MnFeCuCe@C-800 °C, and MnFeCuCe@C-900 °C were synthesized through different thermal annealing procedures based on the MnFeCuCe@C-20% sample. A systematic investigation was conducted on the EMW absorption characteristics and associated electromagnetic parameters of the MnFeCuCe@C composites. The real part of the complex permittivity (*ε′*) represents charge storage capability, and the imaginary part (*ε″*) corresponds to dielectric loss capability. As shown in Fig. S11A and C, both the dielectric loss tangent and maximum dielectric loss values of the 4 samples decreased with increasing frequency. The magnitude of dielectric loss across the measured frequency band followed the sequence: MnFeCuCe@C-700 °C < MnFeCuCe@C-800 °C < MnFeCuCe@C-1,000 °C < MnFeCuCe@C-900 °C. This trend in dielectric loss magnitude suggests that MnFeCuCe@C-900 °C and MnFeCuCe@C-1,000 °C possess stronger dielectric loss capabilities, and corresponding data in Fig. S11A and C indicate that they also exhibit superior charge storage capabilities. We attribute this enhancement to controlled calcination temperatures, which influence metal atom diffusion and coalescence. This process induces lattice imperfections within high-entropy metal particles, promoting the formation of increased dipoles around defect sites. Concurrently, multiple polarization relaxation mechanism is activated, resulting in additional Cole–Cole semicircles and prolonged polarization relaxation times, as evidenced in Fig. 4A1 to A4. The semicircles in the Cole–Cole plot of MnFeCuCe@C are consistent. This indicates that the lattice distortion induced by the heat treatment can form more defect sites, under the action of a high-frequency alternating electric field; each sample exhibits consistent dipole polarization loss around the defects. However, compared to MnFeCuCe@C-700 °C, the other 3 samples display longer tailing, indicating more pronounced conductive loss effects [47]. High metal loading tends to suppress the electrical conductivity of MnFeCuCe@C composites, whereas temperature compensation exerts a positive effect on electron migration rates. MnFeCuCe@C-900 °C and MnFeCuCe@C- 1,000 °C exhibit similarly high conductivity (Fig. 4B and C). In contrast, MnFeCuCe@C-700 °C and MnFeCuCe@C-800 °C possess lower conductivity values of 1.86 and 1.63 mS·cm^−1^, respectively. The *ε*_c_″ curves are closely related to the conductivity of the samples (Fig. 4D). This can be attributed to the reduced graphitization of MnFeCuCe@C and the massive formation of nonconductive high-entropy oxides, and the imperfect electronic structure leads to lower EMW dissipation. In summary, the superior EMW performance of MnFeCuCe@C is closely linked to its appropriate conductivity values [44]. Furthermore, the *ε*_p_″ curves of MnFeCuCe@C-900 °C and MnFeCuCe@C-1,000 °C display 2 distinct relaxation peaks within the 5 to 13 GHz frequency band, which is attributed to the interfacial polarization between high-entropy metal particles and carbon fibers, and the equivalent dipoles induced by localized electron migration inside MnFeCuCe@C composites. Both MnFeCuCe@C-700 °C and MnFeCuCe@C-800 °C exhibit lower *ε*_p_″ values across the entire tested frequency range; additionally, their polarization loss fluctuation patterns are nearly identical, indicating that these samples possess comparably low and almost equivalent polarization effects [48].

The multidimensional comprehensive evaluation of EMW absorption performance focuses on 3 key parameters: absorption intensity, matching thickness, and EAB. As evidenced in Fig. 4F1 to F4 and Fig. S12, the RL_min_ values exhibit important enhancement with increasing heat treatment temperature: MnFeCuCe@C-700 °C (RL_min_ is −10.12 dB with a thickness of 2.50 mm), MnFeCuCe@C-800 °C (RL_min_ is −11.76 dB with a thickness of 2.0 mm), MnFeCuCe@C-900 °C (RL_min_ is −20.19 dB with a thickness of 2.33 mm), and MnFeCuCe@C-1,000 °C (RL_min_ is −81.44 dB with a thickness of 1.52 mm). The maximum absorption bandwidth in the high-frequency region shows a consistent increasing trend (Fig. 4G and Fig. S13). At a thickness of 1.6 mm, the EAB values reach 5.13 GHz for MnFeCuCe@C-900 °C and 4.82 GHz for MnFeCuCe@C-1,000 °C. Furthermore, increasing the calcination temperature reduces the matching thickness required for optimal performance. The thickness corresponding to the RL_min_ value decreases progressively from 2.50 mm (700 °C) to 1.52 mm (1,000 °C). This reduction in matching thickness is attributed to the synergistic effects of multiple electromagnetic loss mechanisms. Figure 4H and Figs. S14 and S15 illustrate that the variation of impedance matches with frequency and thickness for MnFeCuCe@C-700 °C, MnFeCuCe@C-800 °C, MnFeCuCe@C-900 °C, and MnFeCuCe@C-1,000 °C. Benefiting from the strong electron transfer effects of the surface magnetic metal particles in the MnFeCuCe@C-1,000 °C absorber, its impedance matching value predominantly falls within the optimal range of 0.8 to 1.2 for thicknesses ranging from 1 to 5 mm. In addition, this also indicates that the hierarchical porous structure effectively enhances the impedance matching between the surface of MnFeCuCe@C-1,000 °C and free space, creating many channels for the effective propagation of incident EMWs. This strengthens the multiple scattering effect, facilitating the maximum penetration of EMWs into the interior of MnFeCuCe@C-1,000 °C, where they are absorbed and attenuated.

Driven by the dual attenuation mechanisms of dielectric loss and magnetic loss (Fig. 4I and Fig. S11C and D), despite its lower dielectric loss tangent compared to MnFeCuCe@C-900 °C, optimal impedance matching results in the strongest wave absorption loss for MnFeCuCe@C-1,000 °C. The complex permeability of MnFeCuCe@C absorbers is determined by the real part (*μ′*), representing magnetic energy storage capability, and the imaginary part (*μ″*), indicating magnetic loss capability. MnFeCuCe@C-900 °C demonstrates the highest *μ′* values across the entire frequency range, indicating enhanced electromagnetic energy storage capacity. It exhibits only marginally higher *μ″* values than the other materials within the high-frequency region (14 to 18 GHz), signifying slightly better electromagnetic dissipation capability in that specific band. As illustrated in Fig. 4J, thermal treatment enhances EMW attenuation capacity. However, for MnFeCuCe@C-900 °C, neither the optimal absorption bandwidth nor the EAB values corresponding to the RL_min_ values synergize effectively with its attenuation capability (*α*). Therefore, increasing the calcination temperature allows for better tailoring of the electromagnetic response profile, leading to optimized EMW absorption intensity at high frequencies. Furthermore, the contributions from eddy current loss are comparable across all materials (Fig. S16).

### Evaluation of practical applications for MnFeCuCe@C nanocomposites

**Fig. 5.**
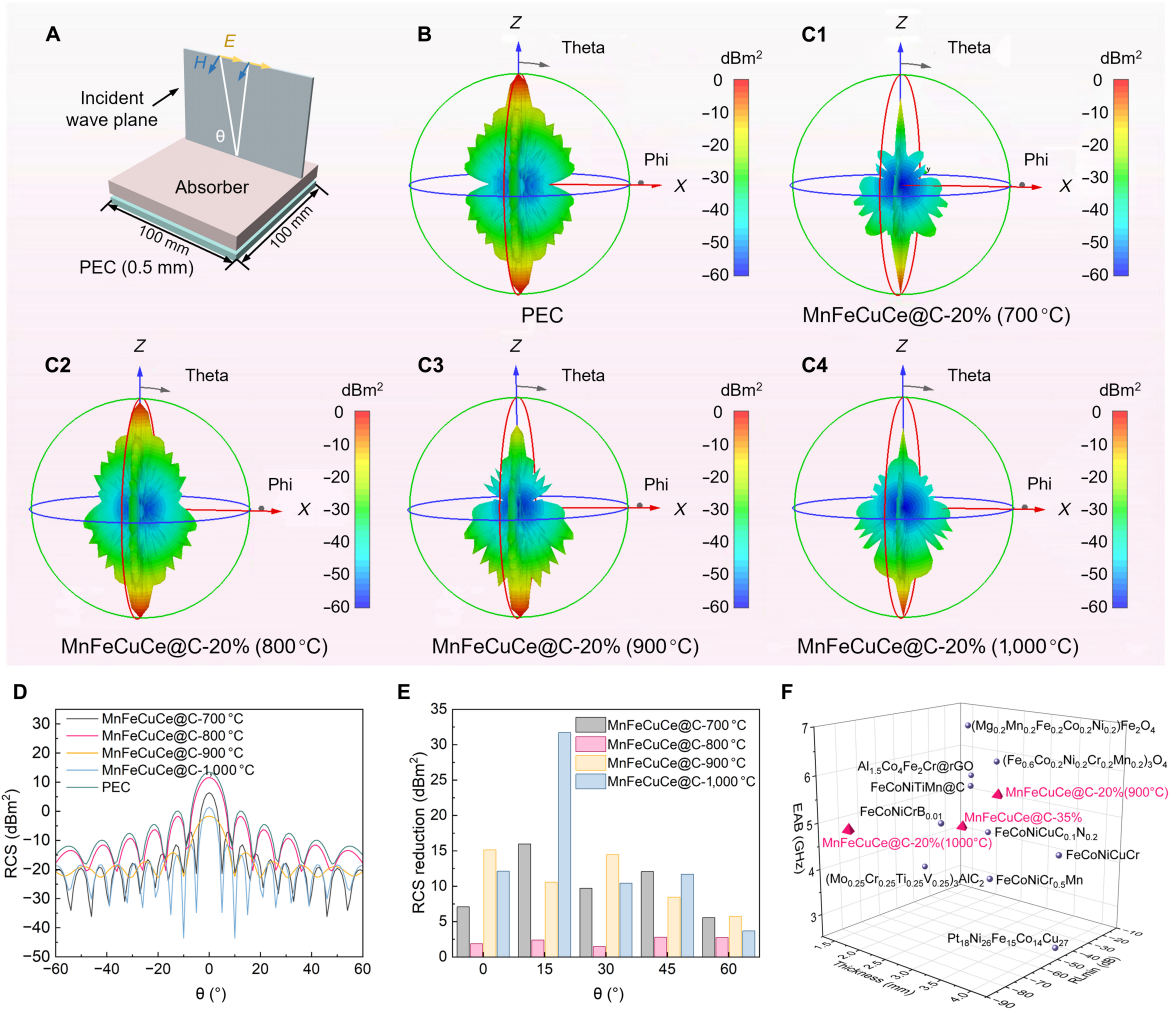
(A) The model of RCS simulation. Three-dimensional RCS plots of (B) the PEC substrate and (C1 to C4) the PEC substrate covered with different EMW absorption materials. (D) RCS plot in the cartesian coordinate system under certain detecting angles. (E) RCS reduction values of MnFeCuCe@C-700 °C, MnFeCuCe@C-800 °C, MnFeCuCe@C-900 °C, and MnFeCuCe@C-1,000 °C. (F) Comparison of EAB/RL_min_/thickness. Detailed performance information and references are listed in Table S1.

RCS is an important parameter for radar detection and identification, and stealth and anti-stealth technology; RCS simulation serves to predict and emulate an electromagnetic attenuation performance of absorbers in real-world conditions [49]. As shown in Fig. 5A, the CST Studio Suite 2023 software is employed to simulate the RCS values of the perfect electric conductor (PEC) plate (size: 100 × 100 × 0.5 mm^3^) and absorber-coated PEC plates, where the direction of plane wave is along the negative *Z*-axis and θ is the incident angle. Figure 5C1 to C4 display the 3-dimensional (3D) RCS patterns of the MnFeCuCe@C absorbers. It can be observed from the images that all samples exhibit varying degrees of reduction in reflected signal intensity compared to PEC (Fig. 5B) at their corresponding matching thicknesses and frequency, which demonstrates their remarkable EMW absorption capability. Additionally, in Fig. 5D and Fig. S17, the 2D RCS fluctuation plots have an incident angle from −60° to 60°. The curves for MnFeCuCe@C-900 °C and MnFeCuCe@C-1,000 °C exhibit greater stability. MnFeCuCe@C-1,000 °C achieves its maximum RCS reduction (31.72 dBm^2^) at 15°, while MnFeCuCe@C-900 °C peaks (15.14 dBm^2^) at 0° (Fig. 5E), demonstrating excellent electromagnetic attenuation performance, which makes them suitable for complex radar detection scenarios with diverse far-field emission bands. Finally, Fig. 5F provides a comparative analysis between this work and existing multicomponent microwave absorbers. The MnFeCuCe@C composites demonstrate a larger EAB and minimal reflection loss, and the meticulously designed MnFeCuCe@C-1,000 °C exhibits a superior specific reflection loss value (Eq. S9) of −267.89 dB mg^−1^/wt% compared to other EMW absorbing materials, indicating superior EMW absorption performance. These results suggest that they are promising next-generation candidates for practical EM wave absorbing applications.

## Conclusion

In summary, an ultra-facile in situ approach facilitated the growth of [Zn(pz)₂]*_n_* complexes onto the surface of cotton fibers. Through optimization of the dynamic balance of element content and carbonization temperature, high-entropy metal particles effectively resolved impedance mismatch in carbon fiber matrices; meanwhile, in-depth investigation of the linkage relationship between microstructure and EM parameters revealed that the magnetic–dielectric synergy within MnFeCuCe nanoparticles and at MnFeCuCe/C heterointerfaces constitutes the determining factors for high-performance EMW absorption. At ultralow loading of 20 wt%, the MnFeCuCe@C composites achieved a wide EAB of 5.13 GHz (achieved at 900 °C, a thickness of 1.6 mm) and exceptional absorption intensity of −81.44 dB (achieved at 1000 °C, a thickness of 1.52 mm), demonstrating promising EMW attenuation capabilities. Furthermore, RCS simulations verified the practical applicability of the absorber. From an industrial development perspective, its rapid synthesis, environmental benignity, and cost efficiency underscore good potential for EMW absorption applications, providing profound insights for designing advanced electromagnetic absorbers.

## Materials and Methods

Additional information about the materials and methods used for this work is available in the Supplementary Materials.

## Data Availability

The authors do not have permission to share data.
