# Supplementary material for: Large-Scale Facile Synthesis of Biomass Fibers and High-Entropy Metal Hierarchical Porous Carbon toward Enhanced Electromagnetic Absorption
Source: Research (Wash D C). 2025 Sep 16;8:0868. doi: 10.34133/research.0868 (PMC12437104; doi:10.34133/research.0868)
Supplement: Supplementary 1 — Materials and Methods Figs. S1 to S17 Table S1 [file research.0868.f1.docx]

**Supporting Information**

**Large-Scale Facile Synthesis of Biomass Fibers and High-Entropy Metal Hierarchical Porous Carbon Toward Enhanced Electromagnetic Absorption**

Peiyu Cui,^#1^ Pengbo Zou,^#1^ Yifan Kang,^1^* Xiang Yan,^1^ Xin Zhou,^1^ BoKun Wang,^1^ Fan Wu,^2^* Shibing Pan,^3^ Jiacheng Ma, ^1^* and Wenhuan Huang,^1^*

#These authors contribute equally to this work.

Email: huangwenhuan@sust.edu.cn

^1^ Key Laboratory of Chemical Additives for China National Light Industry, College of Chemistry and Chemical Engineering, Shaanxi University of Science and Technology, Xi’an 710021, P. R. China.

^2^ Department of Chemistry, School of Science, Tianjin University, Tianjin, 300072, China.

^3^ Shandong Nonmetallic Material Institute, 250031, China.

**1. Experimental Section**

**1. 1 Materials**

The raw cotton fiber was obtained from a local marketplace. Zinc chloride (ZnCl_2_), Pyrazole, Ammonium hydroxide (NH_3_·H_2_O), Manganese acetate tetrahydrate (Mn(CH_3_COO)_2_·4H_2_O), Iron chloride solution (FeCl_3_), Cupric Acetate Monohydrate (Cu(CH_3_COO)_2_·H_2_O), Cerium tetranitrate (Ce(NO_3_)_4_), Methanol. All the above drugs were used directly without further purification.Experimental sections.

**1.2 Preparation of Cotton@Zn-pz**

First, 0.6 g of pyrazole was dissolved in 10mL deionized water, 1 g cotton fiber was immersed in pyrazole solution and sonicated for 30 min, so that the cotton absorbed the pyrazole solution and evenly distributed in the cotton and dried the water, then 0.4 g ZnCl_2_ and NH_3_·H_2_O(0.2 mL) were dissolved in 10 mL of deionized water and stirred evenly, and the cotton obtained from the previous step was immersed in ZnCl_2_ solution and ultrasonicated for 30 min, and Cotton@Zn-pz was obtained after drying.

**1.3 Preparation of MnFeCuCe@CZp**

Taking MnFeCuCe@CZp-5% as an example, Mn(CH_3_COO)_2_·4H_2_O (12.7 mg), Cu(CH_3_COO)_2_·H_2_O (10 mg), FeCl_3_ (8.4 mg), and Ce(NO_3_)_4_ (20 mg) were completely dissolved in a methanol (20 mL), and the dried MnFeCuCe@CZp was immersed in the metal solution for 30 min and then dried to obtain dry MnFeCuCe@CZp-5%. Similar procedure steps were followed to obtain MnFeCuCe@CZp-20% and MnFeCuCe@CZp-35% respectively. MnFeCuCe@CZp-20% is obtained by simultaneously expanding increasing the mass of the metal salt by a factor of five. MnFeCuCe@CZp-35% is obtained by simultaneously expanding increasing the mass of the metal salt by a factor of ten.

**1.4 Preparation MnFeCuCe@C**

Under the protection of N_2_ atmosphere, cotton, MnFeCuCe@C-5%, MnFeCuCe@C-20% and MnFeCuCe@C-35% were obtained by heating at 1000 °C for 2 h with 5 ℃ min^-1^. The as-prepared MnFeCuCe@C-20% was directly calcined in a N_2_ atmosphere at 700, 800 ,900 or 1000°C for 2 h with a heating rate of 5°C min^-1^. The calcined materials were denoted as MnFeCuCe@C-700°C, MnFeCuCe@C-800°C, MnFeCuCe@C-900°C and MnFeCuCe@C-1000°C, respectively.

**2. Characterizations**

The crystal structure of the samples was investigated using an X-ray diffractometer (Bruker Advance D8, Germany, λ = 0.154 nm, 4° min^-1^). The morphology of the crystalline powders was characterized using field emission scanning electron microscopy (FEI Verios 460, USA). X-ray photoelectron spectroscopy was realized on a Thermo Fisher Scientific Escalab 250 Xi. Raman spectra of the samples were obtained by Renishaw Invia Raman microscope, UK. The specific surface area of the samples was determined by adsorption and desorption of the samples using a Kantar Autosorb-iQ at 77 K. The wave-absorbing powder samples were mixed well according to the mass ratio of 4:16 of wave-absorbing powder samples to paraffin wax, and pressed to form annular pellets with an inner diameter of 2.0 mm and an outer diameter of 7.0 mm for the testing of electromagnetic parameters. The reflection loss (RL) values were calculated by putting the measured data into programmed formula according transmission line theory. An electrochemical workstation (CHI760E) is used to test the electrochemical impedance spectra (EIS) of MnFeCuCe@C

sample in the 15 wt% KOH solution. The EIS test is carried out with a scan amplitude of 5 mV and frequencies ranging 10^6^ Hz to 1 Hz. The electrochemical tests adopt a three-electrode system. A sample-loaded conducting glass is used for the working electrode, and a platinum metal sheet and saturated calomel electrode (SCE) are used as auxiliary and reference electrodes, respectively. The samples were prepared as follows: first, 10 mg of sample powder, 50 μL of Nafion solution and 200 μL of deionized water were uniformly mixed. Subsequently, 35 μL of the mixed solution was added dropwise onto a conducting glass with size of 2 ×2 cm^2^. Finally, these samples were dried at 60 °C for 10 minutes.

# 3. Data analysis

In these equations, Z_in_ and Z_0_ are the input impedance of absorbing materials and the impedance of free space, d is the thickness, c is the speed of light, f is the frequency, $\varepsilon_{r}$ and $\mu_{r}$are the complex permittivity and permeability, respectively. ^[1]^

$Z_{in}=Z_{0}\sqrt{\frac{\mu_{r}}{\varepsilon_{r}}}\tan h\left[ j\frac{2\pi fd}{c}\sqrt{\varepsilon_{r}\mu_{r}} \right]$ (Equation S1)

$\mathrm{RL}\left( dB \right)=20\log\left| \frac{\left( Z_{in}-Z_{0} \right)}{\left( Z_{in}+Z_{0} \right)} \right|$ (Equation S2)

𝜀_𝑝_” and 𝜀_𝑐_” are the dielectric loss contributed by polarization relaxation and charge transport, respectively, which can be obtained according to Debye theory (Eq. S3-5)

$\varepsilon_{c}^{''}=\frac{\sigma}{2\pi f\varepsilon_{0}}$ (Equation S3)

$\varepsilon_{P}^{''}=\frac{\varepsilon_{s}-\varepsilon_{\infty}}{1+\left( 2\pi f \right)^{2}\tau^{2}}\omega\tau={\varepsilon^{''}-\varepsilon}_{c}^{''}$ (Equation S4)

$\varepsilon^{''}=\frac{\varepsilon_{s}-\varepsilon_{\infty}}{1+\left( 2\pi f \right)^{2}\tau^{2}}\omega\tau+ \frac{\sigma}{2\pi f\varepsilon_{0}}={\varepsilon_{P}^{''}+\varepsilon}_{c}^{''}$ (Equation S5)

It is generally recognized that the Cole-Cole semicircle can be explained by the relaxation process, and the relationship between ε′ and ε′′ can be expressed as (Equation S6):

$${(\varepsilon^{'}-\frac{\varepsilon_{s}+\varepsilon_{\infty}}{2})}^{2}+{(\varepsilon'')}^{2}={(\frac{\varepsilon_{s}-\varepsilon_{\infty}}{2})}^{2} \text{ }\text{(Equation }\text{S}\text{6}\text{)}$$

The ability of microwave absorbers to attenuate electromagnetic wave energy is determined by the attenuation constant (*α*), which can be calculated by the following formula (Equation S7):

$\alpha=\frac{\sqrt{2}}{c}\pi f\sqrt{\varepsilon^{''}\mu^{''}-\varepsilon^{'}\mu^{'}+\sqrt{\left( \varepsilon^{''}\mu^{''}+\varepsilon^{'}\mu^{'} \right)^{2}+\left( \varepsilon^{'}\mu^{''}+\varepsilon^{''}\mu^{'} \right)^{2}}}$ (Equation S7)

The criterion of *C_0_* is usually used to evaluate the contribution of eddy current:

$C_{0}=\varepsilon^{''}{{(\mu}^{'})}^{-2} f^{-1}=2\pi{\sigma\mu}_{0}d^{2}$ (Equation S8)

SRL can be expressed as (Equation S9):

$SRL=\frac{\mathrm{RL}_{\min}}{(F\mathrm{ill}\mathrm{ing} \times Thickness)}$ (Equation S9)

# 4. Results and discussions


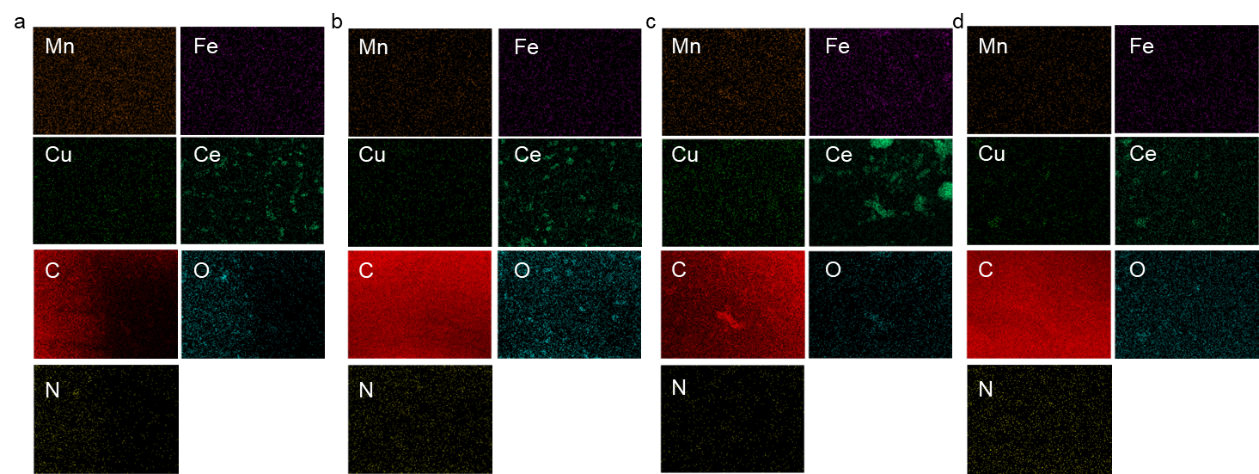


**Figure S1.** The elemental Mapping of **a)** MnFeCuCe@C-700℃, **b)** MnFeCuCe@C-800℃, **c)** MnFeCuCe@C-900℃, **d)** MnFeCuCe@C-1000℃


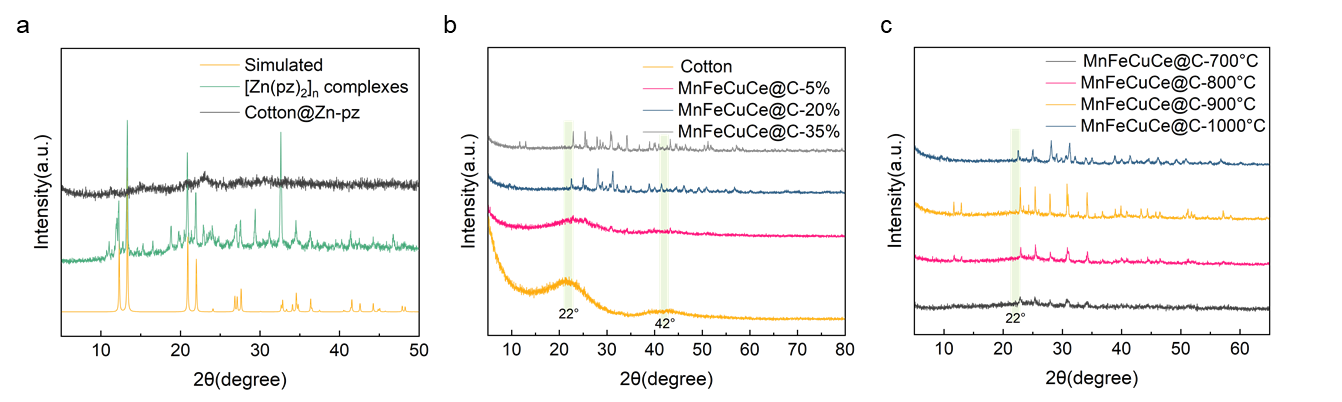


**Figure S2. a)** The XRD patterns of [Zn(pz)_2_]_n_ complexes and Cotton@Zn-pz, **b)** and **c)** the XRD patterns of MnFeCuCe@C.


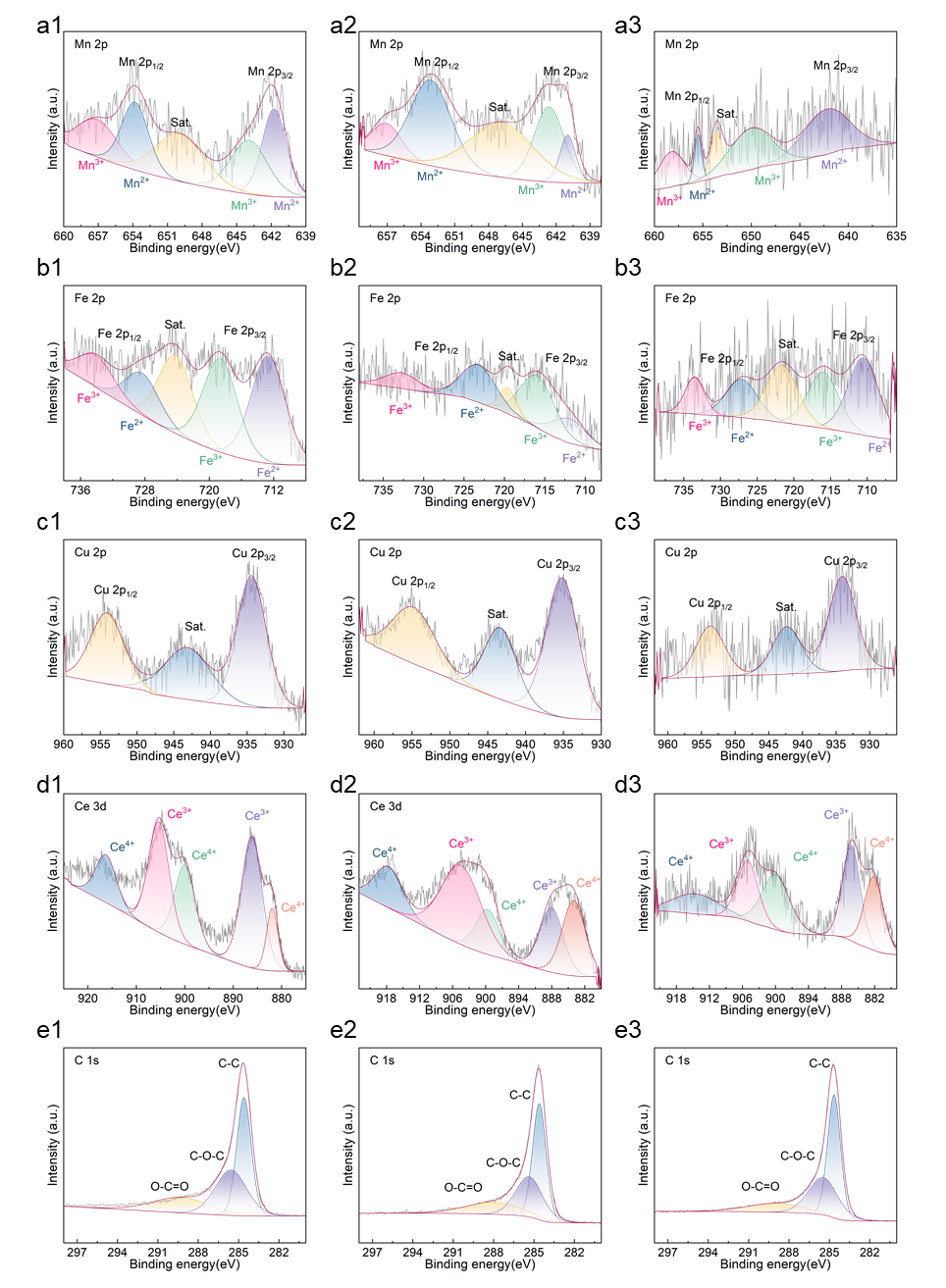


**Figure S3.** The XPS spectra of **a1-e1)** MnFeCuCe@C-700°C, **a2-e2)** MnFeCuCe@C-800°C and **a3-e3)** MnFeCuCe@C-900°C.


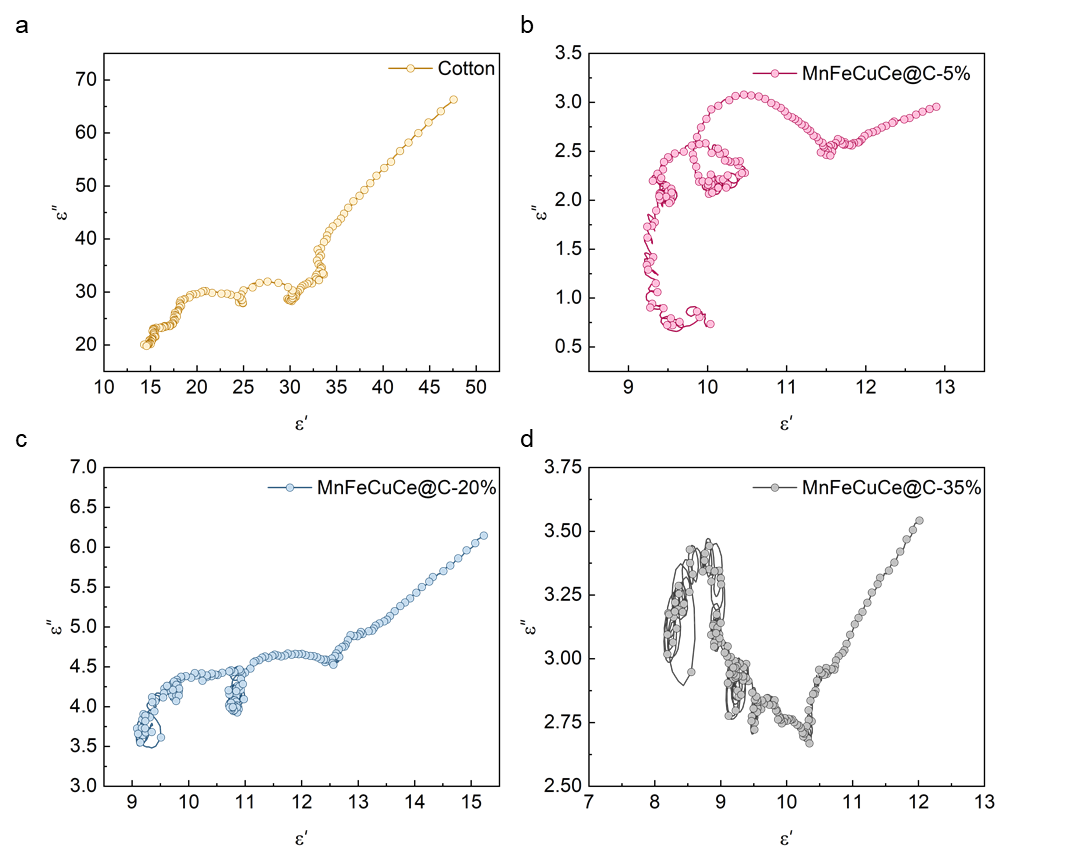


**Figure S4.** Cole–Cole curves (ε′–ε″ plots) of **a)** Cotton, **b)** MnFeCuCe@C-5%, **c)** MnFeCuCe@C-20% and **d)** MnFeCuCe@C-35%.


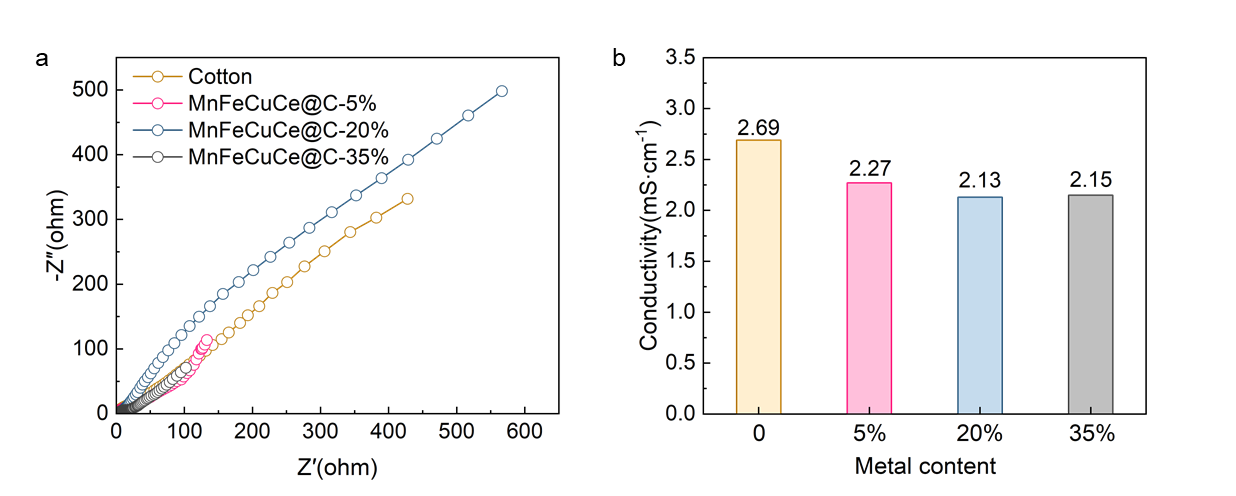


**Figure S5. a)** Nyquist plots. **b)** electrical conductivity.


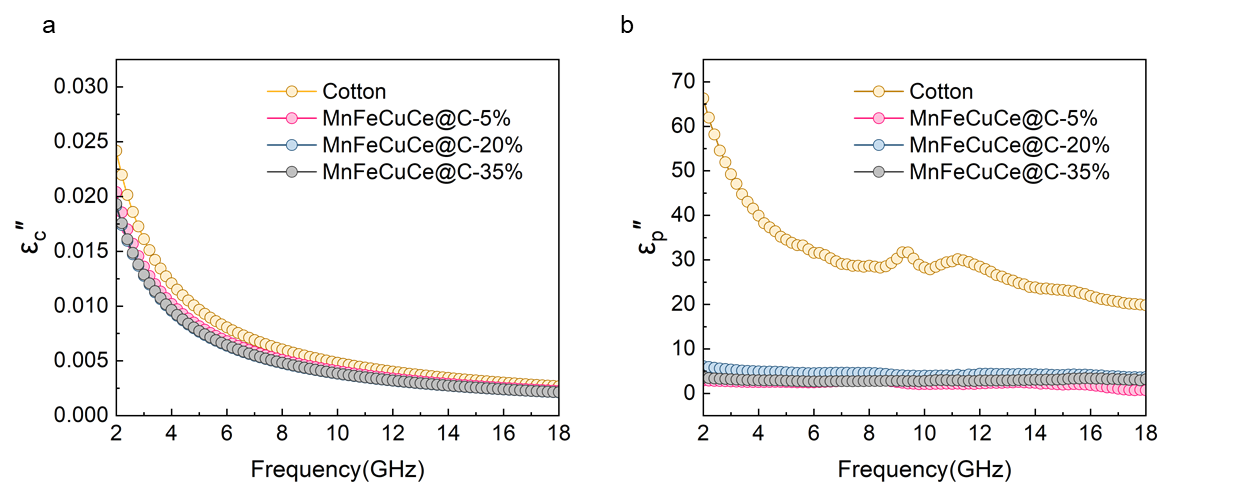


**Figure S6. a)** Conduction loss. **b)** Polarization loss.


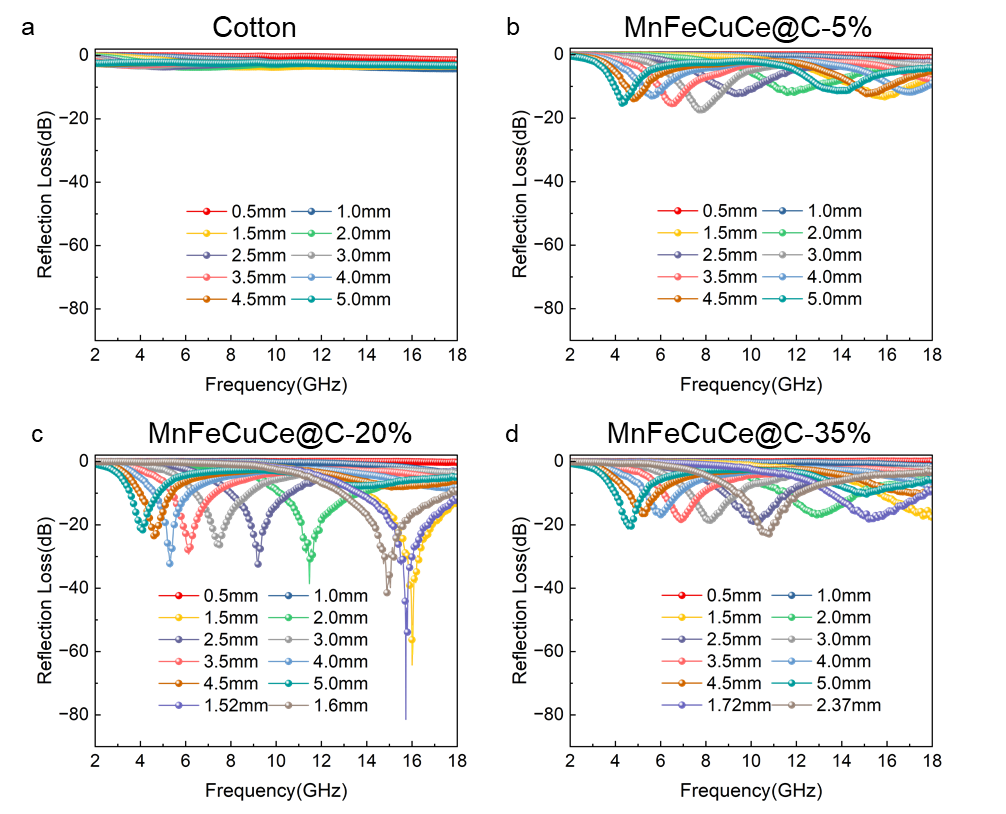


**Figure S7.** The RL plots for **a)** Cotton, **b)** MnFeCuCe@C-5%, **c)** MnFeCuCe@C-20% and **d)** MnFeCuCe@C-35%.


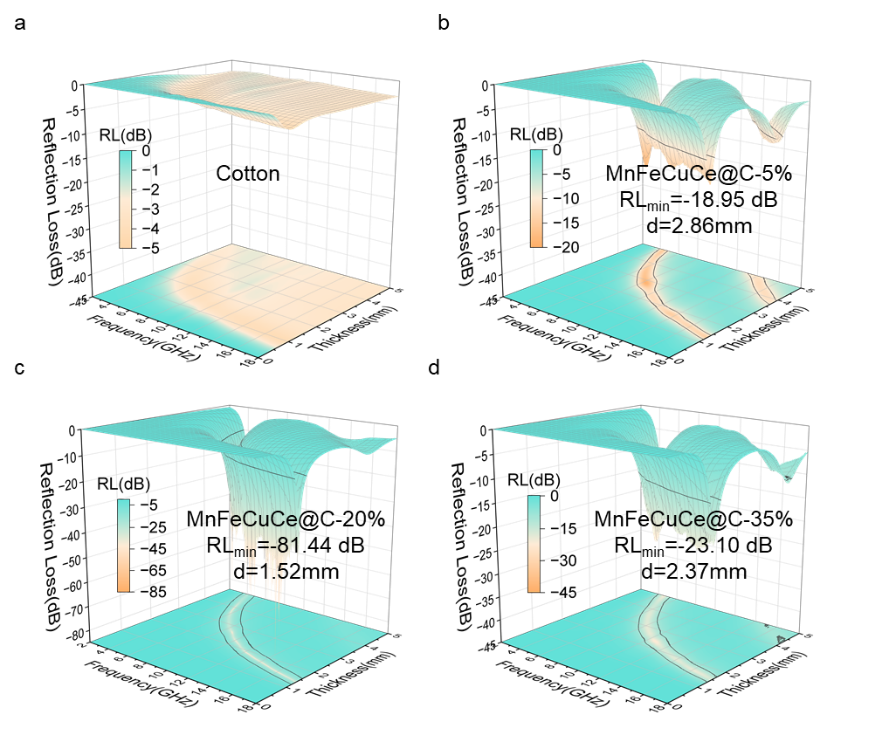


**Figure S8.** The 3D RL maps for **a)** Cotton, **b)** MnFeCuCe@C-5%, **c)** MnFeCuCe@C-20% and **d)** MnFeCuCe@C-35%.


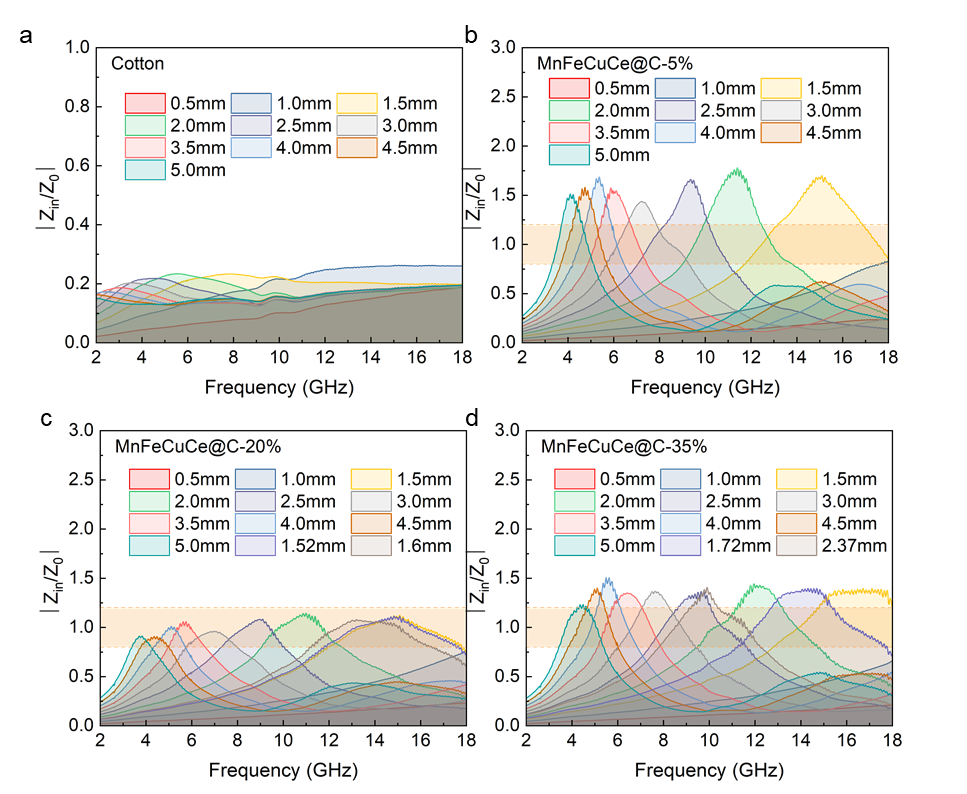


**Figure S9.** The |Z_in_/Z_0_| plots of **a)** Cotton, **b)** MnFeCuCe@C-5%, **c)** MnFeCuCe@C-20% and **d)** MnFeCuCe@C-35%.


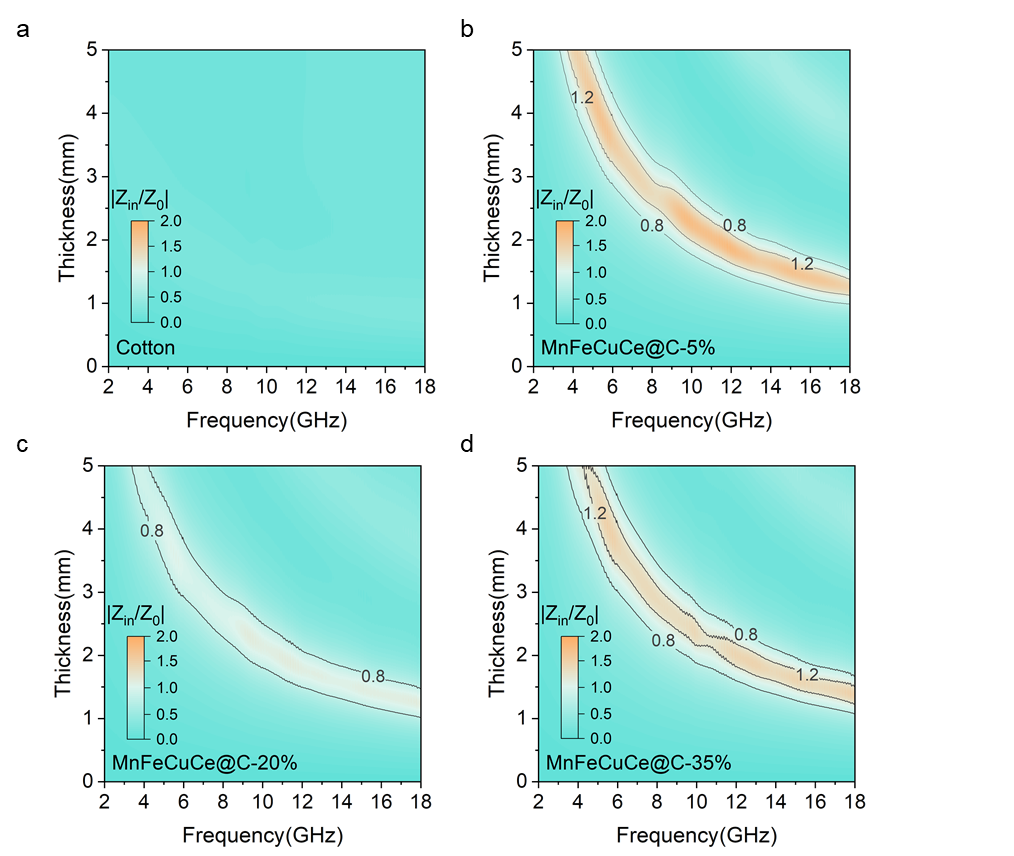


**Figure S10.** The 2D contour map of |Z_in_/Z_0_| for **a)** Cotton, **b)** MnFeCuCe@C-5%, **c)** MnFeCuCe@C-20% and **d)** MnFeCuCe@C-35%.


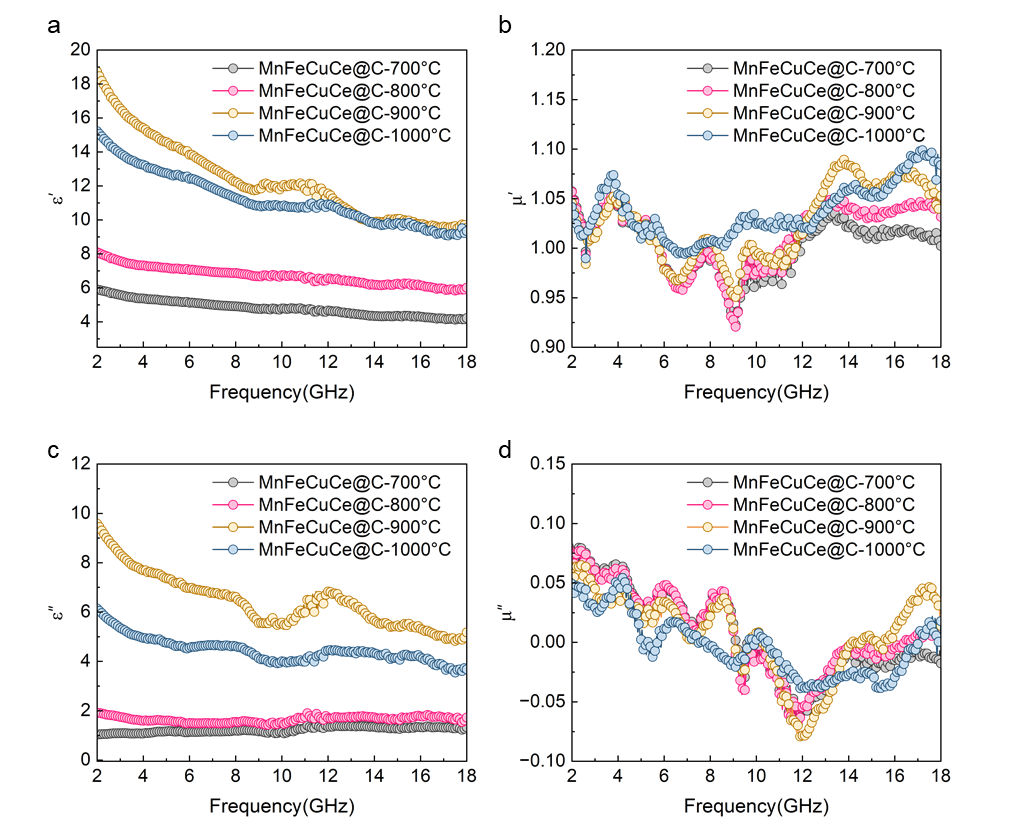


**Figure S11. a)** The ε′, **b)** ε″, **c)** μ' and **d)** μ" values.


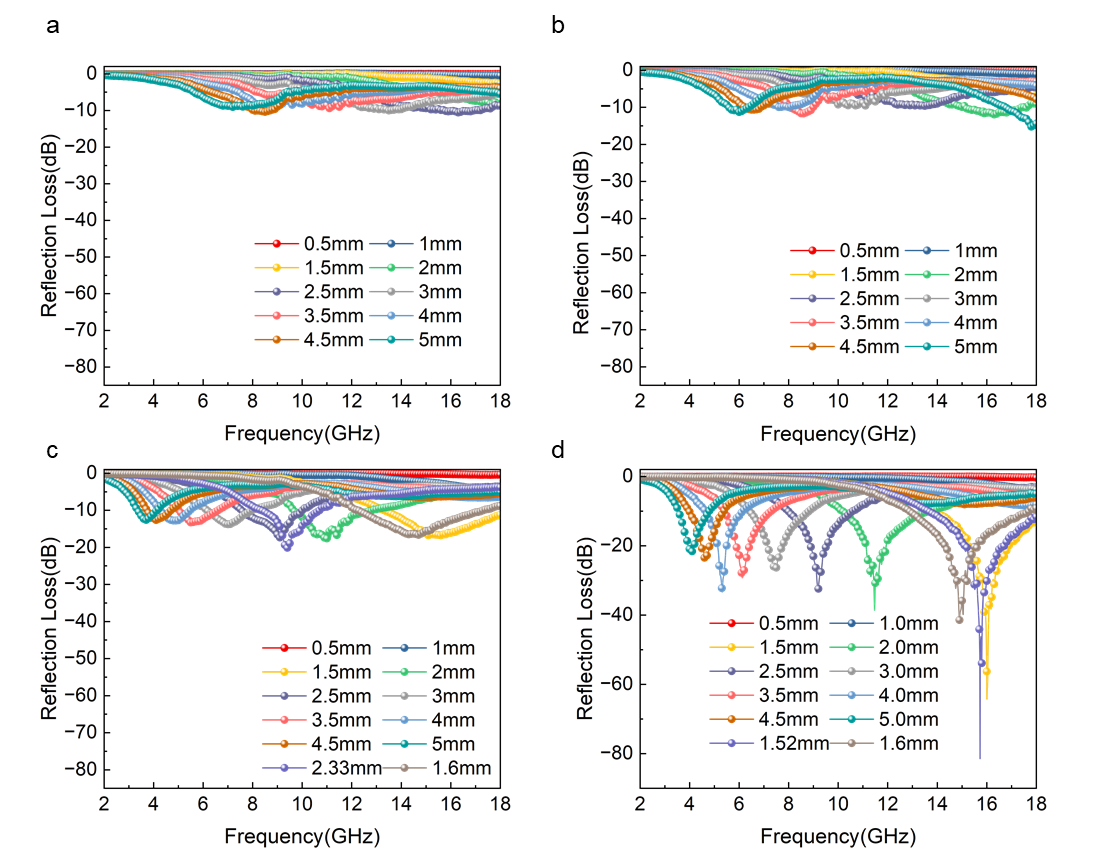


**Figure S12.** The RL plots for **a)** MnFeCuCe@C-700°C **b)** MnFeCuCe@C-800°C, **c)** MnFeCuCe@C-900°C and **d)** MnFeCuCe@C-1000°C.


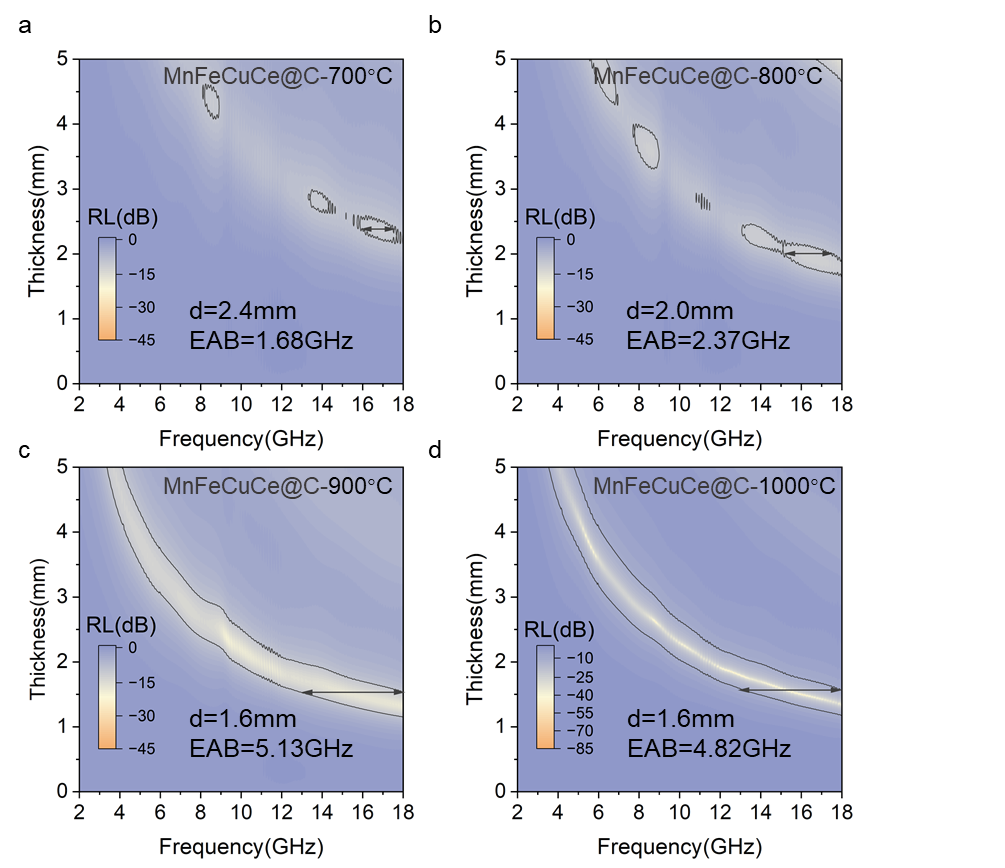


**Figure S13.** 2D RL contour maps for **a)** MnFeCuCe@C-700°C, **b)** MnFeCuCe@C-800°C, **c)** MnFeCuCe@C-900°C and **d)** MnFeCuCe@C-1000°C.


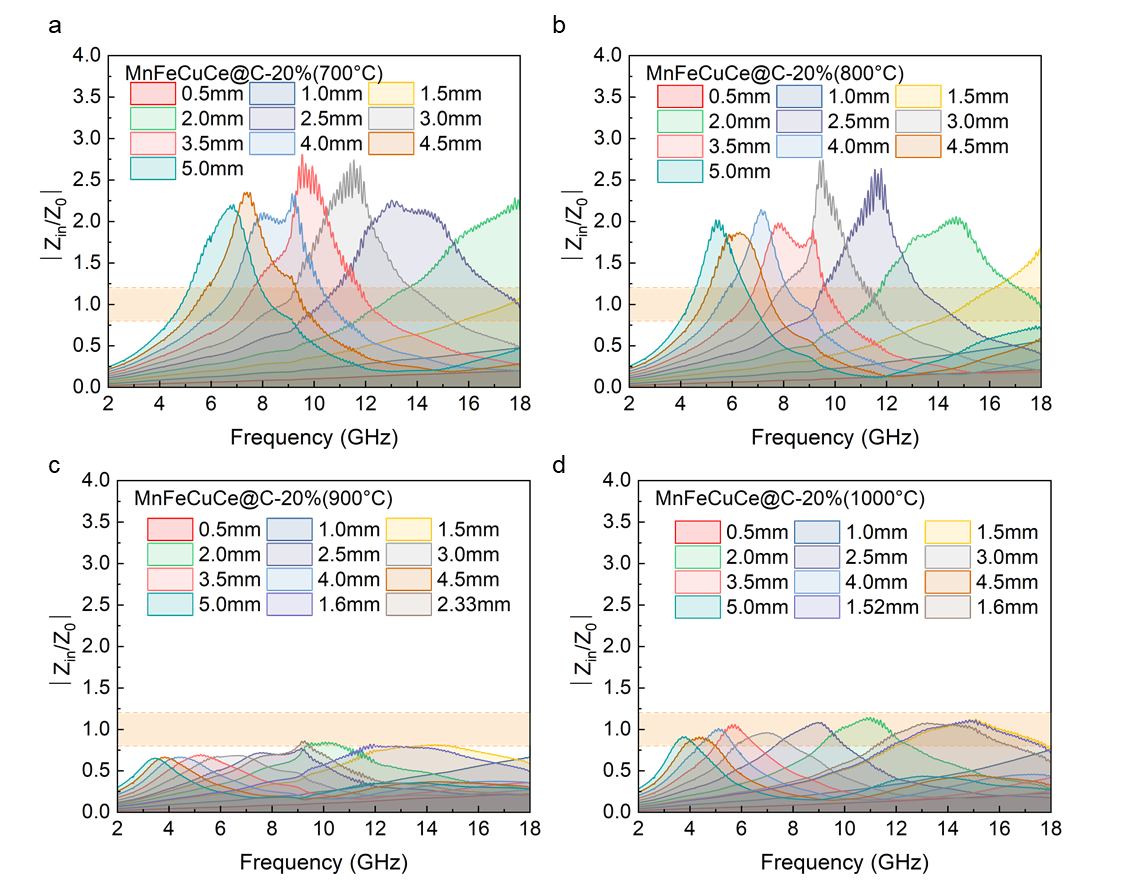


**Figure S14.** The |Z_in_/Z_0_| plots of **a)** MnFeCuCe@C-700°C, **b)** MnFeCuCe@C-800°C, **c)** MnFeCuCe@C-900°C and **d)** MnFeCuCe@C-1000°C.


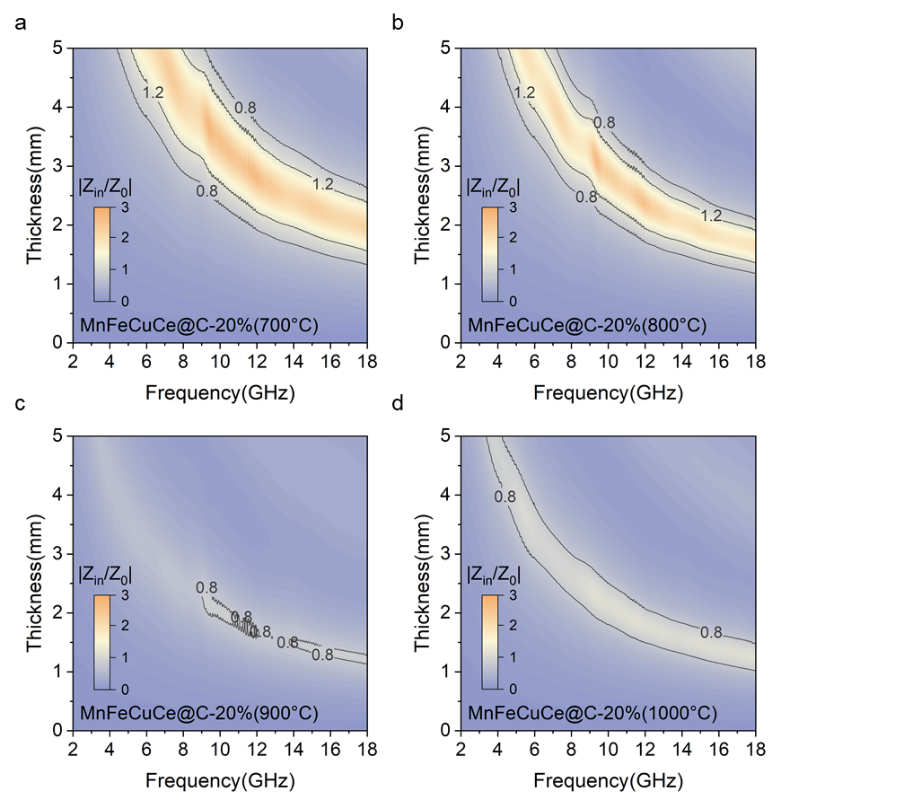


**Figure S15.** The 2D contour map of |Z_in_/Z_0_| for **a)** MnFeCuCe@C-700°C, **b)** MnFeCuCe@C-800°C, **c)** MnFeCuCe@C-900°C and **d)** MnFeCuCe@C-1000°C.


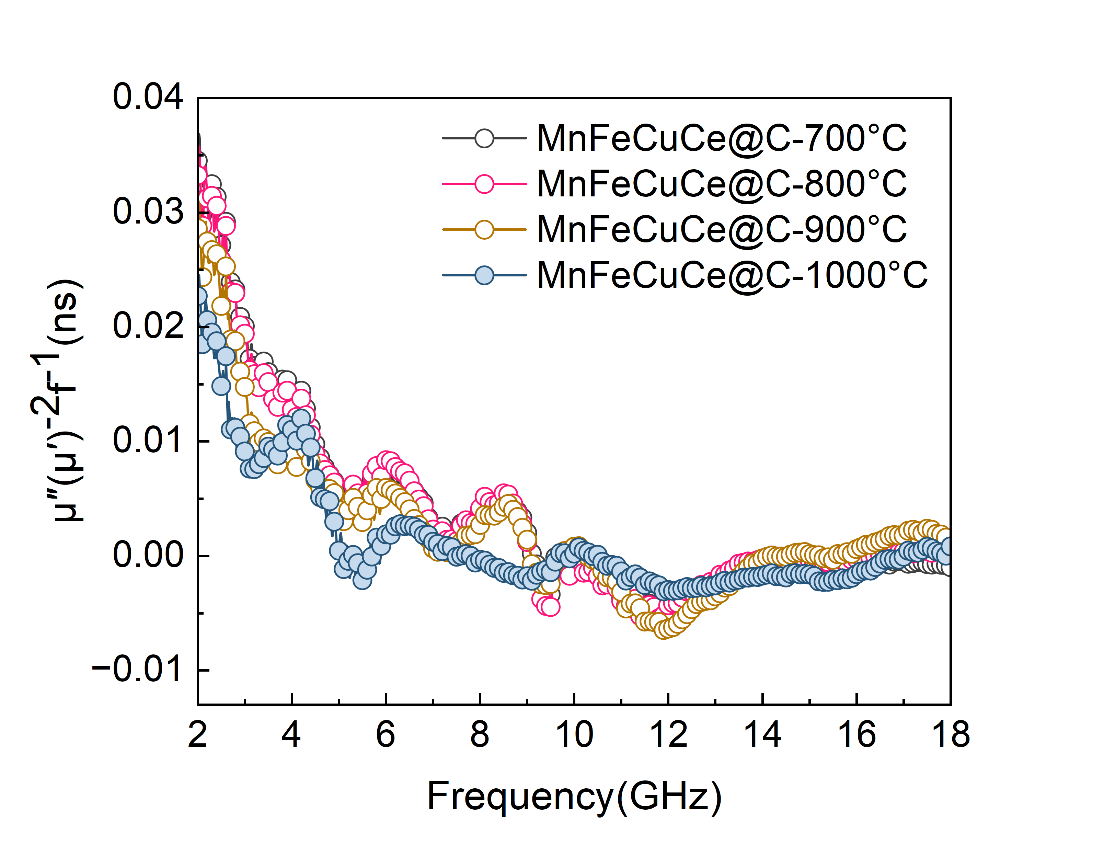


**Figure S16.** *C_0_*-f curve.


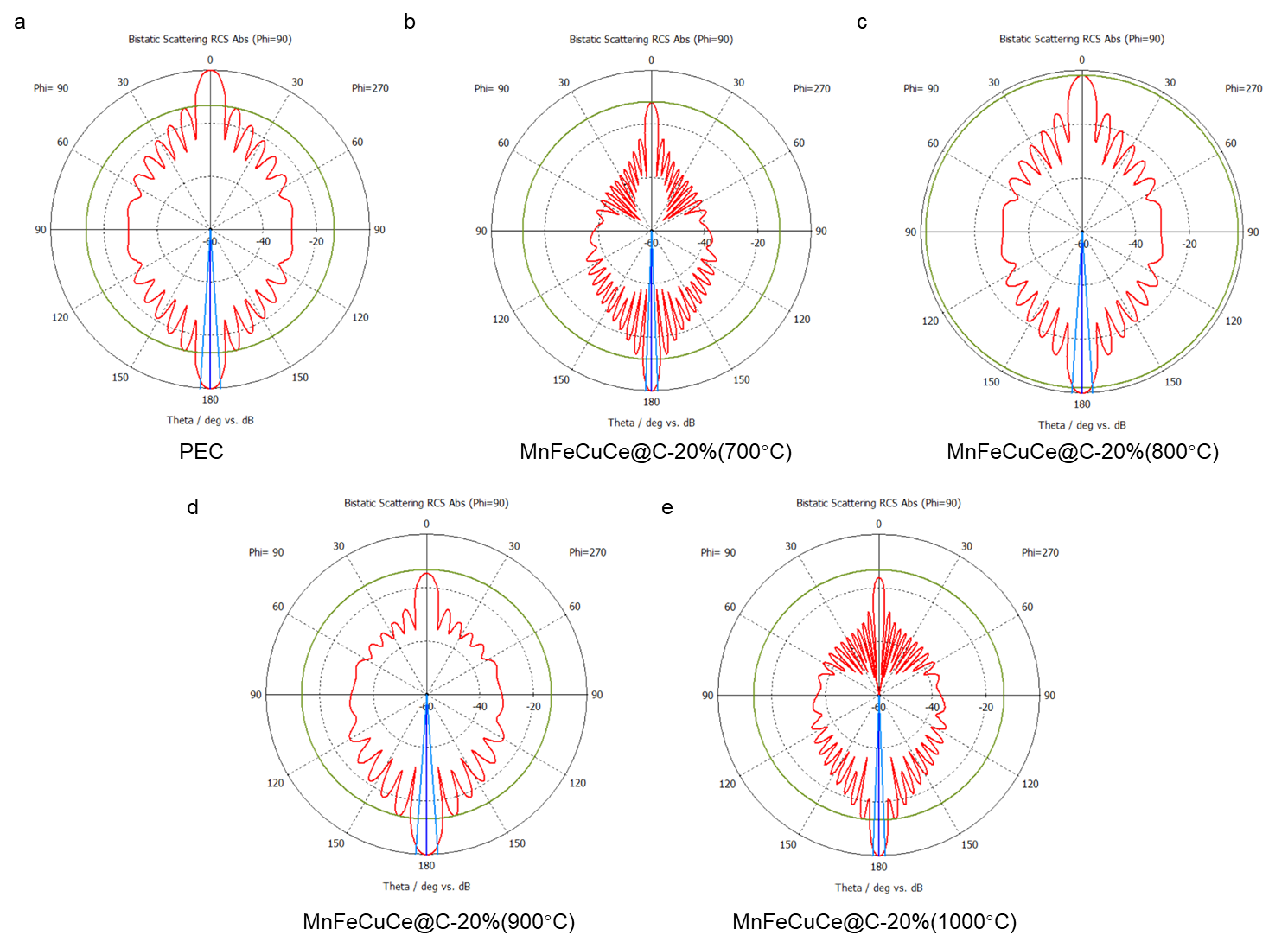


**Figure S17.** RCS values of **a)** PEC, **b)** MnFeCuCe@C-700°C, **c)** MnFeCuCe@C-800°C, **d)** MnFeCuCe@C-900°C and **e)** MnFeCuCe@C-1000°C.

**Table S1.** A series of MnFeCuCe@C composites and other multielement microwave absorbing composites for microwave absorption performance reported in the literatures.

| Samples | RLmin  (dB) | Thickness  (mm) | EAB  (GHz) | SRL  (dB·mm^-1^/wt%) | Filling  (wt%) | Ref. |
| --- | --- | --- | --- | --- | --- | --- |
| Al_1.5_Co_4_Fe_2_Cr@rGO | -17.28 | 1.7 | 5.43 | -14.52 | 70 | 2 |
| FeCoNiTiMn@C | -33.42 | 2.2 | 5.45 | -30.38 | 50 | 3 |
| FeCoNiCuCr | -41.23 | 4.0 | 4.50 | -12.88 | 80 | 4 |
| Pt_18_Ni_26_Fe_15_Co_14_Cu_27_ | -41.80 | 4.0 | 2.50 | / | / | 5 |
| (Mo_0.25_Cr_0.25_Ti_0.25_V_0.25_)_3_AlC_2_ | -45.80 | 1.70 | 3.60 | -35.92 | 75 | 6 |
| FeCoNiCr_0.5_Mn | -57.40 | 3.30 | 4.00 | -24.85 | 70 | 7 |
| (Fe_0.6_Co_0.2_Ni_0.2_Cr_0.2_Mn_0.2_)_3_O_4_ | -34.0 | 2.7 | 6.1 | -20.99 | 60 | 8 |
| (Mg_0.2_Mn_0.2_Fe_0.2_Co_0.2_Ni_0.2_)Fe_2_O_4_ | -35.10 | 2.2 | 6.8 | -22.79 | 70 | 9 |
| FeCoNiCuC_0.1_N_0.2_ | -32.30 | 2.5 | 4.46 | -18.46 | 70 | 10 |
| FeCoNiCrB_0.01_ | -64.50 | 2.66 | 5.08 | -34.64 | 70 | 11 |
| MnFeCuCe@C-20%  (900°C) | -20.19 | 2.33 | 5.13 | -43.33 | 20 | This work |
| MnFeCuCe@C-20%  (1000°C) | -81.44 | 1.52 | 4.82 | -267.89 | 20 | This work |
| MnFeCuCe@C-35% | -23.10 | 1.72 | 4.26 | -67.15 | 20 | This work |

**5. References**

[1] Yang X, Gao W, Chen J, Lu X, Yang D, Kang Y, Liu Q, Qing Y, Huang W. Co-Ni electromagnetic coupling in hollow Mo_2_C/NC sphere for enhancing electromagnetic wave absorbing performance. *Chin. J. Chem.* 2023; 41: 64-74.

[2] Wang S, Zhang W, Zhang Y, Zhao J, Li R, Zhong Y. Effect of reduced graphene oxide on microwave absorbing properties of Al_1.5_Co_4_Fe_2_Cr high-entropy alloys. *Entropy*. 2024; 26(1): 60.

[3] Li Y, Liao Y, Ji L, Hu C, Zhang Z, Zhang Z, Zhao R, Rong H, Qin G, Zhang X. Quinary high‐entropy‐alloy@graphite nanocapsules with tunable interfacial impedance matching for optimizing microwave absorption. *Small*. 2022; 18: 2107265.

[4] Li G, Zhao H, Wang H, Zhou Z, Gao L, Su W, Dong C. Enhanced microwave absorption performances of FeCoNiCuCr high entropy alloy by optimizing particle size dehomogenization. *J Alloys Compd*. 2023; 941:168822.

[5] Wu P, Kong X, Feng Y, Ding W, Sheng Z, Liu Q, Ji G. Phase engineering on amorphous/crystalline γ‐Fe_2_O_3_ nanosheets for boosting dielectric loss and high‐performance microwave absorption. *Adv Funct Mater*. 2024; 34: 2311983.

[6] Qiao L, Bi J, Liang G, Liu C, Yin Z, Yang Y, Wang H, Wang S, Shang M, Wang W. Synthesis and electromagnetic wave absorption performances of a novel (Mo_0.25_Cr_0.25_Ti_0.25_V_0.25_)_3_AlC_2_ high-entropy MAX phase. *J Mater Sci Technol*. 2023; 137: 112-122.

[7] Zhou H, Jiang L, Zhu S, Wang L, Hu Y, Zhang X, Wu A. Excellent electromagnetic-wave absorbing performances and great harsh-environmentresistance of FeCoNiCrxMn high entropy alloys. *J Alloys Compd*. 2023; 936: 168282.

[8] Dai G, Deng R, Zhang T, Yu Y, Song L. Quantitative evaluation of loss capability for in situ conductive phase enhanced microwave absorption of high-entropy transition metal oxides. *Adv Funct Mater*. 2022; 32: 2205325.

[9] Ma J, Zhao B, Xiang H, Dai F, Liu Y, Zhang R, Zhou Y. High-entropy spinel ferrites MFe_2_O_4_ (M=Mg, Mn, Fe, Co, Ni, Cu, Zn) with tunable electromagnetic properties and strong microwave absorption. *J Adv Ceram*. 2022; 11: 754-68.

[10] Jia L, Jiang L, Hu J, Jin J, Yan S, Wu A, Zhang X. Efficient and homogeneous carbonitriding of high-entropy alloys by a mechanochemical process for obtaining coordinated electromagnetic matching. *Acs Appl Mater Inter*. 2023; 15: 58651-58662.

[11] Zhou H, Jiang L, Jia L, Zhu S, Wang L, Wu A, Zhang X. Interstitial boron-doped FeCoNiCr high entropy alloys with excellent electromagnetic-wave absorption and resistance to harsh environments. *J Alloys Compd*. 2023; 959: 170579.
